# 1,2-DCA biodegradation potential of an aquifer assessed in situ and in aerobic and anaerobic microcosms

**DOI:** 10.1186/s40793-024-00650-w

**Published:** 2024-12-18

**Authors:** Ilenia Cruciata, Laura Scirè Calabrisotto, Giovanna Carpani, Lucia Poppa, Alfonso Modica, Andrea Pace, Valentina Catania, Paola Quatrini

**Affiliations:** 1 Department of Biological, Chemical and Pharmaceutical Sciences and Technologies (STeBiCeF), University of Palermo, Palermo, Italy; 2 Environmental and Biological Laboratories, Eni S.p.A, San Donato Milanese, MI Italy; 3 Eni Rewind S.p.A, Fano, PU Italy; 4 Environmental Laboratory Services, Eni Rewind S.p.A, Priolo Gargallo, SR Italy; 5 Present Address: Department of Earth and Marine Sciences (DiSTeM), University of Palermo, Palermo, Italy; 6 Present Address: Department of Engineering, University of Palermo, Palermo, Italy

**Keywords:** Chlorinated hydrocarbons, Bioremediation, Reductive dehalogenase, Haloalkane dehalogenase, Groundwater Microbiota

## Abstract

**Background:**

1,2-dichloroethane (1,2-DCA) biodegradation can occur through aerobic or anaerobic pathways that can be exploited in bioremediation strategies. Bioremediation interventions are site specific and generally based on anaerobic pathways, nevertheless expanding knowledge on proper conditions favoring the biodegradation and especially on 1,2-DCA degrading microorganisms is crucial. In this work the intrinsic biodegradation potential of an aquifer impacted by Chlorinated Aliphatic Hydrocarbons (mainly 1,2-DCA) was evaluated by characterizing the aquifer microbiome across space and time and by setting up biostimulation treatments in microcosms under different aerobic and anaerobic conditions, in parallel.

**Results:**

The microbial profiling of the aquifer revealed noticeable alpha and beta diversity across the sampling sites within the aquifer and strong fluctuations over time. Surprisingly both the anaerobic and aerobic biostimulation treatments led to the successful removal of 1,2-DCA in microcosms, the enrichment of known 1,2-DCA degraders and the detection of reductive or hydrolytic dehalogenases. *Ancylobacter* and *Starkeya* were enriched in aerobic microcosms. *Desulfovibrio* and *Desulfuromonas*, known as perchloroethylene degraders, were enriched in anaerobic microcosms, suggesting they could be yet unknown 1,2-DCA respirers.

**Conclusions:**

Our results demonstrate the occurrence of both aerobic and anaerobic bioremediation potential in the aquifer despite its negative redox potential. Due to the feasibility of direct oxidation with oxygen insufflation, we propose that an enhanced bioremediation strategy based on direct oxidation of 1,2-DCA could be applied to the contaminated aquifer as an ecofriendly, efficient and cost-effective approach as an alternative to anaerobic biodegradation.

**Supplementary Information:**

The online version contains supplementary material available at 10.1186/s40793-024-00650-w.

## Introduction

1,2-dichloroethane (1,2-DCA) is a persistent, toxic and probably carcinogenic chlorinated aliphatic hydrocarbon (CAH) commonly found in contaminated aquifers. Bioremediation strategies can be applied for the clean-up of 1,2-DCA by exploiting and enhancing the known biodegradation pathways of this contaminant carried out by bacteria [1–3].

1,2-DCA biodegradation can occur under both anaerobic and aerobic conditions. Under anaerobic conditions 1,2-DCA can be used as an electron acceptor in a respiratory process called reductive dechlorination, that mainly consists in dichloroelimination, yielding ethene [4]. Reductive dechlorination is mediated by reductive dehalogenases and is carried out by obligate Organohalide Respiring Bacteria (OHRB) such as *Dehalococcoides*, *Dehalobacter*, and *Dehalogenimonas* [5–7] or facultative OHRB such as *Desulfitobacterium* [4]. Recently, a Compound-Specific Isotope Analysis (CSIA) in combination with Biological Molecular Tools (BMTs) revealed that *Geobacter* sp. was the main responsible for significant 1,2-DCA dichloroelimination processes in groundwater under anaerobic conditions [8]. Reductive dehalogenases are encoded by several diverse genes generally referred to as *rdh* whose sequences are difficult to associate to a range of substrates [9]. Anaerobic 1,2-DCA degradation can also occur by anaerobic cometabolic reactions fortuitously mediated by an enzyme or cofactor, such as highly reducing corrinoids and other cofactors such as vitamin B_12_, coenzyme F430, or hemes produced during microbial metabolism of another compound. This pathway is carried out by methanogens, acetogens, or sulfate-reducing bacteria as reported for the 1,2-DCA cometabolic degradation carried out by a homoacetogen *Acetobacterium* sp. [10]. During this process, the microorganism does not yield growth or energy, therefore a primary substrate is required to support the growth of microorganisms and sustain the cometabolic process [11]. Cometabolism is thought to be less significant than halorespiration due to slower rates of reaction [10]. Moreover, the contribution of cometabolic processes in reductive dechlorination reactions can be difficult to address, as it occurs as a fortuitous transformation of a compound by enzymes used for other purposes [12]. Under aerobic conditions, 1,2-DCA is used as a carbon and energy source in a hydrolytic pathway mediated by the haloalkane dehalogenase DhlA encoded by the *dhlA* gene [13], as in *Xanthobacter* and *Ancylobacter*, or in a monooxygenase-mediated pathway, as in *Pseudomonas* and *Polaromonas* [14–17]. Both pathways, which are globally referred to as Direct Aerobic Oxidation (DAO) [13], lead to the formation of chloroethanol, which is further oxidized to monochloroacetate and subsequently dehalogenated to glycolic acid. Aerobic cometabolism of 1,2-DCA consists in the fortuitous oxidation of the chlorinated molecule by microbial nonspecific enzymes produced for the uptake of other compounds used as growth substrates, such as methane, ammonia and toluene [2]. In most cases, these enzymes are monooxygenases that can accidentally attack non-growth substrates due to their broad substrate range, cleaving carbon-halogen bonds or facilitating the spontaneous decomposition of the chlorinated molecule in aqueous medium through the formation of labile structures [12]. Similarly to anaerobic cometabolism, this process does not benefit microorganisms in terms of energy or growth. Therefore, cometabolic processes require the presence of substrates usually used by microorganisms to support their growth [2, 18].

When investigating the feasibility of site-specific bioremediation strategies or the occurrence of natural biodegradation processes (natural attenuation), laboratory-scale microcosms are a crucial tool for testing multiple substrate types and the impact of amendments such as nutrients, vitamins, electron acceptors or donors on biodegradation processes and microbial growth rates. Microcosms are usually set up using site matrices as inocula and periodically monitored by chemical and biological analysis to assess the occurrence of a degradation process [3, 4, 19]. Column studies can also be carried out as a further step, providing additional information on hydraulic factors, if required, and test materials for permeable barriers or bioreactors [20]. When investigating reductive dechlorination in laboratory-scale systems, the chemicals added include vitamin B_12_, an important cofactor in reductive dehalogenases [21], and an electron donor source, depending on the OHRB. Most OHRB such as *Dehalococcoides* [5], *Dehalogenimonas* [22], *Dehalobacter* [23] and *Desulfitobacterium* use hydrogen as the electron donor for 1,2-DCA degradation, but *Desulfitobacterium* also uses lactate and formate as electron donors [10]. H_2_ can also be the result of fermentation processes by non-dechlorinating members of the microbial communities, therefore various substrates, such as lactate, acetate, formate, butyrate, ethanol, glucose [24, 25] or slow-release H_2_ sources such as molasses [3], have been tested. Recently, low-cost slow-release substrates such as poly-β-hydroxybutyrate [26] were also successfully applied in the bioremediation of CAHs [27], including 1,2-DCA [20].

Lab-scale systems investigating direct aerobic processes do not generally require the addition of substrates beside the CAH used by microorganisms as growth and energy source, but they require a supply of oxygen [13]. However, only few CAHs can be biodegraded by DAO and knowledge on strains, pathways and necessary conditions for CAHs direct aerobic degradation is limited, thus hindering DAO-based bioremediation approaches [13]. In aerobic cometabolic processes, beside oxygen, appropriate growth substrates (co-substrates) stimulating the production of oxygenase enzymes must be supplied, such as ethane, butane, ammonia [2, 28].

Currently, 1,2-DCA bioremediation approaches mainly focus on anaerobic reductive dechlorination or aerobic cometabolic processes [2]. Although it is already known that bacteria capable of DAO can coexist with anaerobic dechlorinating bacteria in the same aquifer [29], we demonstrated that both conditions result in CAHs biodegradation in microcosm.

In this work the intrinsic biodegradation potential of an aquifer contaminated by CAHs (mainly 1,2-DCA, vinyl chloride and 1,1-dichloroethylene) and heavy metals was investigated for the first time *(i)* by metagenomic profiling of the autochthonous microbial communities throughout the study site and during time in the most contaminated monitoring well and *(ii)* by evaluating the response of these communities to both anaerobic and aerobic biostimulation treatments in microcosm. The results provide knowledge on microorganisms and their catabolic pathways in the perspective of bioremediation strategies to be applied to CAHs contaminated aquifers.

## Materials and methods

### Description of the contaminated area

The contaminated site investigated in this study is part of a large industrial area located in southern Italy and is characterized by a significant and long-term groundwater (GW) contamination by chlorinated solvents, predominantly 1,2-DCA and vinyl chloride (VC). The concentrations of these compounds have been detected many orders of magnitude above the limits established by Italian law (3 µg/L for 1,2-DCA and 0.5 µg/L for VC, D. Lgs. 152/2006), up to 320 mg/L for 1,2-DCA and 21 mg/L for VC (see Supplementary Material 1).

**Fig. 1:**
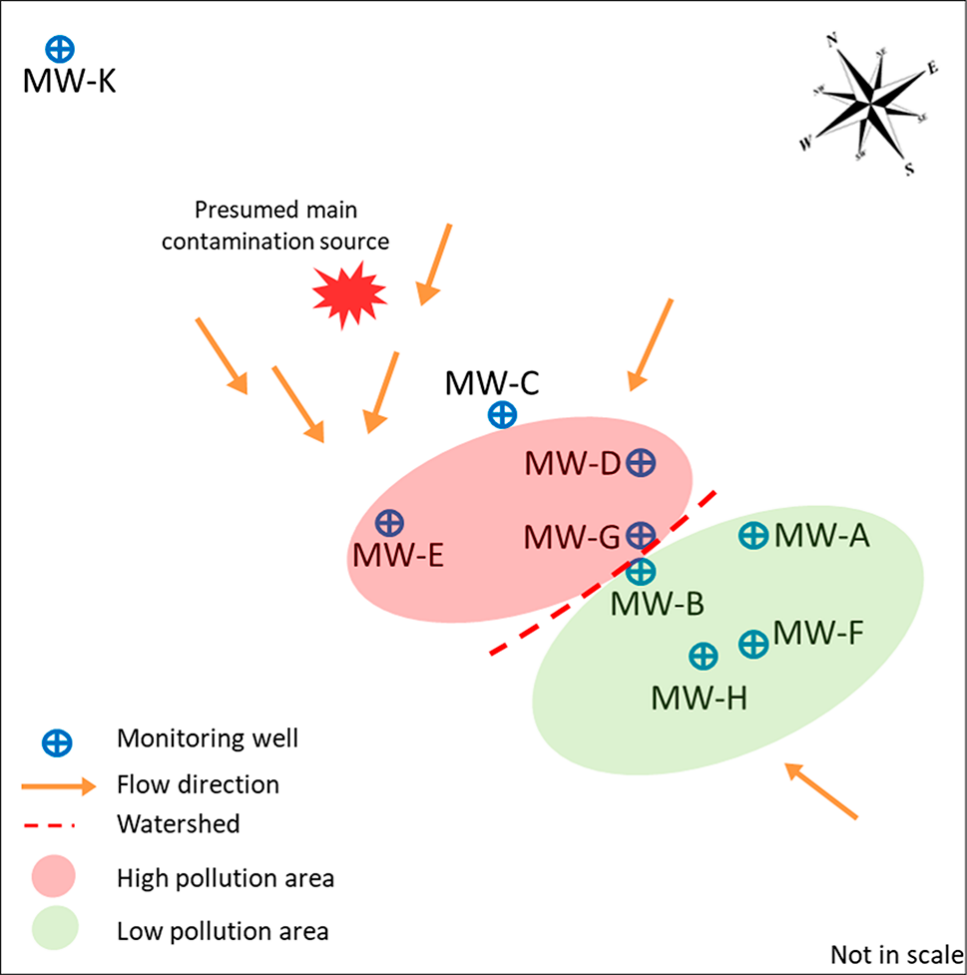
Map of the site. Schematic map of the contaminated area showing the localization of monitoring wells considered in this study, the groundwater flow direction, the presumed main contamination source and the distribution of the pollution

In the contaminated area (approximately 3000 m^2^), eight monitoring wells (MWs), named MW-A, -B, -C, -D, -E, -F, -G and -H, are utilized for GW sampling, in order to periodically characterize chemical-physical properties and contamination levels of the aquifer (Fig. 1).

The main groundwater flow goes from north-east to south-west. GW collected from MW-D, MW-E, and MW-G is characterized by high concentrations of chlorinated compounds, mainly 1,2-DCA (320 mg/L, 25 mg/L, and 130 mg/L, respectively). Taking into account the aquifer flow paths, the aforementioned MWs intercept the main channel from north-east and might collect a main pollution source and significant flow rates. In contrast, the area of MW-A, MW-B, MW-F, and MW-H is generally characterized by low concentrations of 1,2-DCA and seems to be interested by lower flow rates. Moreover, this low pollution area seems to be hydraulically separated from the high pollution one by an underground watershed, thus justifying the significant difference in the distribution of 1,2-DCA contamination in the study area (Fig. 1).

### Groundwater sampling

GW samples were collected in a time period from 2019 to 2022 from the eight MWs placed in the contaminated area. Upstream this area the MW-K represents a sampling point to recover groundwater not interested by chlorinated solvents contamination, that was used as a control for the subsequent investigations (Fig. 1). MW-D, that historically showed the deepest 1,2-DCA contamination, was selected to collect GW for three years consecutively (hereinafter referred as years 1, 2 and 3).

GW was withdrawn from each MW at 11 m depth using a submersible pump and GW samples were collected in sterilized N_2_-fluxed dark glass bottles with no head-space to minimize oxygen contamination. After collection, the samples were preserved on ice and in the dark and transported to the laboratory, where they were processed within 48 h.

For each sampling event, GW chemistry parameters were assessed both in the field and in the laboratory. GW quality parameters including oxidation-reduction potential (ORP), dissolved oxygen (DO), temperature, pH, and electrical conductivity (EC) were measured in the field using a multimeter sensor. Analyses of 1,2-DCA, VC and other CAHs (chloroform, 1,1-dichloroethylene, 1,2-dichloroethylene, 1,1-dichloroethane, 1,1,2-trichloroethane) were performed in the laboratory by Gas Chromatograph/Mass Spectrometry in accordance with EPA method 8260D 2018 or APAT CNR IRSA method 5150 29/2003. The concentrations of chlorides, fluorides, sulfates, and phosphates were determined using EPA method 9056A 2007. Ammonium was measured using APAT CNR IRSA method 4030 A2 C 29/2003. Various metals were detected using EPA method 6020B 2014.

### Groundwater filtration and DNA extraction

Water biomass was collected by filtering 1 L of each GW sample in duplicate. A preliminary filtration was carried out for year 1 samples using two different systems: sterile 0.2 μm-pore-sized surfactant-free cellulose acetate (SFCA) membranes (NALGENE^®^) using a vacuum pump or sterile 0.2 μm-pore-sized polyethersulfone (PES) membranes using a Sterivex™ system (EMD Millipore Corporation, Billerica, MA, USA) in order to compare their extraction efficiency. After the filtration, each membrane was sheared from its own system and stored at -20 °C.

Metagenomic DNA extraction from the filters was performed using the QIAamp^®^ DNA Stool Mini kit (QIAGEN, Hilden, Germany) according to the manufacturer’s recommendations with slight modifications at the initial and final steps. Specifically, each filtering membrane was scraped, broken into pieces and transferred into a conical tube; InhibitEx buffer (2 mL) was added to the fragmented membrane and the sample was mixed by vortexing. After incubation at 95 °C for 5 min, the suspension was centrifuged (15,000 rcf) for 1 min, and the supernatant was applied to the remaining steps of the proposed protocol. The resulting metagenomic DNA was eluted in 50 µL of UltraPURE™ DNase/RNase-free distilled water (GIBCO^®^), checked for quality and concentration with a NanoDrop^®^ ND-1000 spectrophotometer (NanoDrop Technologies, LLC Wilmington, DE) and stored at -20 °C until further processing.

### Automated ribosomal intergenic spacer analysis (ARISA)

The diversity of the indigenous microbial communities of the GW samples was initially investigated using automated ribosomal intergenic spacer analysis (ARISA) as described by Catania et al. [30]. Briefly, metagenomic DNA from each sample was used as a template in PCR amplifications with the universal primers ITSF/ITSReub targeting the 16S–23S rRNA intergenic transcribed spacers (ITS) [31]. ITSReub was labelled with the phosphoramidite dye HEX (6-carboxy-1,4-dichloro-2,4,5,7-tetra-chlorofluorescein). Amplified ITS fragments (1–3 µL of PRC product) were separated by capillary electrophoresis on an ABI PRISM^®^ 310 Genetic Analyzer (Applied Biosystems, Foster City, CA, USA) according to manufacturer’s instructions. Electropherograms were analyzed using the Peak Scanner™ software v1.0 (Applied Biosystems). The fluorescence threshold was set at 50 relative fluorescence units (RFU). Fragments between 50 and 1000 bp in length were considered and exported into spreadsheets for subsequent analysis. A bin size of 5 bp was employed to minimize inaccuracies in the ARISA profiles. Alpha and beta diversity were estimated using the PAST software v2.17c [32].

### 16S rRNA gene metagenomic sequencing and data processing

The metagenomic high-throughput sequencing of 16S rRNA gene was used to define the taxonomic profile of the autochthonous microbial communities for each GW sample. As described by Corsino et al. [26], metagenomic DNA obtained from each GW sample was used as template to amplify the V3-V4 hypervariable region of the 16S rRNA gene, for the simultaneous detection of Bacteria and Archaea, using Pro341F/Pro805R primers [33]. Amplicon sequencing was carried out on an Illumina-MiSeq^®^ platform with 300 bp paired-end reads. Reads denoising and filtering were performed using Qiime2 tools version 2019.4. Taxonomy was assigned using trained operational taxonomic units (OTUs) at 99% from GreenGenes database version 13–8. 16S rRNA gene sequences obtained in this study were deposited into the NCBI short reads archive (SRA) database under BioProject ID PRJNA1134153 (http://www.ncbi.nlm.nih.gov/bioproject/1134153) (BioSample accession numbers: SAMN42394718 - SAMN42394732).

### OHRB-specific 16S rRNA gene amplification

In order to detect the presence of OHRB genera in the microbial communities from the most 1,2-DCA contaminated GW, amplifications by nested PCR of phylogenetic biomarkers specific of *Dehalococcoides*, *Dehalogenimonas*, *Dehalobacter*, *Desulfitobacterium*, and *Desulfuromonas* were performed on metagenomic DNA extracted from GW sample from MW-D for each sampling event, as proposed in Löffler et al. [34]. An initial amplification of the microbial community 16S rRNA genes was performed using universal primers 27F/1492R Supplementary Material 2 reports the list of primers used). The reaction mixtures were prepared in a final volume of 30 µL and contained 0.75 U of DreamTaq™ DNA polymerase (ThermoFisher Scientific™), 1X DreamTaq Buffer (ThermoFisher Scientific™), a 200 µM concentration of dNTPs mixture (Invitrogen), a 0.2 µM concentration of each primer and 2–50 ng of total DNA as template. Cycle conditions consisted of an initial denaturation at 95 °C for 3 min, followed by 30 cycles of amplification (95 °C for 30 sec, 50 °C for 30 sec, 72 °C for 1 min) and a final elongation at 72 °C for 15 min. For the nested PCR step, 1:5 dilutions of the 16S rRNA gene amplicons obtained were used as templates for PCR with OHRB-specific primer pairs (Supplementary Material 2). The PCR reaction mix composition was essentially the same as the first step, except that the reaction volume was reduced to 20 µL and the final concentration varied for each primer pairs (i.e., 0.4 µM for Dhc1f/Dhc264r and Desulfo16sF3/Desulfo16sR5, 0.5 µM for Dhb477f/Dhb647r and BL-DC-142f/BL-DC-1351r, 0.25 µM for Dsm16Sf/Dsm16Sr). PCR conditions were as described earlier, except that the annealing temperatures for any individual primer pair varied as reported in the Supplementary Material 2.

### Microcosm setup

Microcosm experiments under anaerobic and aerobic conditions were conducted to assess the presence of a biodegradation activity by stimulating the dechlorinating component of the autochthonous bacterial communities of the study area. GW samples collected from MW-D in years 3 and 2 were used to inoculate anaerobic and aerobic microcosms, respectively. Microcosms were prepared in 20 mL glass vials sealed with screw caps with PTFE-faced silicone septa (Phenomenex^®^) in triplicate for each condition. Anaerobic microcosms were set up with 50% (vol/vol) GW and BTZ culture medium [25] to a final volume of 12 mL. The following conditions were realized (Table 1): microcosms containing medium, GW and lactate amendment (1 mM, LAC^*+*^); microcosms containing medium and GW without additive amendment (LAC^−^); abiotic control microcosms containing medium and sterilized GW (AN-AB). 1,2-DCA 100 ppm was added to the BTZ medium as electron acceptor. Anaerobic microcosms were set up in an anaerobic glove box under an atmosphere saturated with filter-sterilized N_2_ and all media and solutions were bubbled with filter sterilized N_2_. The last step of glove box conditioning was performed using a filter-sterilized gas mixture of N_2_/CO_2_/H_2_ (80%/15%/5%) [4]. Anaerobic microcosms were incubated statically in the dark, at room temperature until analysis. Aerobic microcosms were set up with 50% (vol/vol) GW and mineral salt medium (MSM) [35] to a final volume of 12 mL. The following conditions were realized (Table 1): microcosms containing medium, GW and no additive amendment (OX); microcosms containing medium, GW and a cosubstrate (CO); abiotic control microcosms containing medium and sterilized GW (AE-AB). 1,2-DCA 100 ppm was added to the MSM medium as sole carbon and energy source in OX microcosms. A volatile hydrocarbons mixture (1% ethane, 1% methane, 0.01% C_4_-C_6_) was syringe-injected into sealed CO and abiotic control microcosms as a cosubstrate, in addition to 100 ppm 1,2-DCA. Aerobic microcosms were incubated on a rotary shaker at 150 rpm at room temperature in the dark until analysis. GW for abiotic control microcosms was previously treated with 0.1% sodium azide (NaN_3_) as a bacteriostatic agent [8]. At the end of each planned incubation time, the microcosms were sacrificed in triplicate for chemical monitoring. After thirty days of incubation, three replicates of each microcosm were filtered, DNA was extracted and analyzed as described for GW samples.

**Table 1:** Names and composition of anaerobic and aerobic microcosms

Microcosm	Components				
*Anaerobic microcosms*	Medium	Groundwater (% vol/vol)	Contaminant	Sterilizing agent	Added substrate
LAC^+^	BTZ	50	1,2-DCA 100 ppm	-	Sodium-lactate 1 mM
LAC^−^	BTZ	50	1,2-DCA 100 ppm	-	-
AN-AB (abiotic control)	BTZ	50	1,2-DCA 100 ppm	NaN_3_ 0.1%	Sodium-lactate 1 mM
*Aerobic microcosms*					
OX	MSM	50	1,2-DCA 100 ppm	-	-
CO	MSM	50	1,2-DCA 100 ppm	-	Volatile short chain HC mixture 5% vol/vol
AE-AB (abiotic control)	MSM	50	1,2-DCA 100 ppm	NaN_3_ 0.1%	Volatile short chain HC mixture 5% vol/vol

LAC^*+*^: anaerobic microcosms amended with lactate; LAC^−^: anaerobic microcosms not amended with lactate; AN-AB: anaerobic control microcosms with sterilized groundwater; OX: aerobic microcosms without additive amendment; CO: aerobic microcosms containing a cosubstrate; AE-AB: aerobic control microcosms with sterilized groundwater. BTZ: anaerobic culture medium; MSM: aerobic mineral salt medium; HC: hydrocarbon; HC mixture: 1% ethane, 1% methane, 0.01% C_4_-C_6_

### Chemical monitoring of microcosms

Chemical monitoring of 1,2-DCA removal in all microcosms and in aerobic enrichment cultures was performed by gas chromatography - mass spectrometry (GC/MS) by headspace sampling. A 7000 C GC/MS/MS Triple Quad (Agilent Technologies) GC/MS with an Agilent 19,091 S-433UI column (30 m × 0.25 mm diameter × 0.25 μm thickness) with helium carrier gas and equipped with an autosampler was used. Prior to the headspace sampling, the microcosm bottles were incubated 10 min at 30 °C with shaking. 250 µL headspace samples were injected. A split mode with a ratio of 5:1 was used with an injection temperature of 270 °C. The analysis program consisted of an initial GC temperature hold of 40 °C for 0.2 min, followed by an increase to 240 °C at a rate of 10 °C/min. The 1,2-DCA detection limit was approximately 1–2 µg/L. Calibration curves were set up with standard solutions of 1,2-DCA (02562-5ML, Sigma Aldrich) in distilled water (1, 5, 10, 25, 50, 100 ppm). 1,2-DCA concentration was calculated using the Agilent MassHunter Qualitative Data Analysis software.

### Search for reductive and haloalkane dehalogenase genes in microcosms

Degenerated and specific primers (Supplementary Material 2) designed on the sequences of known reductive dehalogenase genes were used to search for *rdh* genes in metagenomic DNAs extracted from anaerobic microcosms. PCR reactions were performed in a total volume of 20 µL containing 0.5 U of DreamTaq™ DNA polymerase (ThermoFisher Scientific™), 1X DreamTaq Buffer (ThermoFisher Scientific™), 0.2 mM dNTPs mixture (Invitrogen), 0.2 to 0.6 µM of forward and reverse primers and 1–10 ng of template DNA. PCR conditions used were as follows: an initial denaturation at 95 °C for 3 min; [95 °C for 30 sec, annealing (Supplementary Material 2) for 30 sec, 72 °C for 1 min] X 30 cycles; a final extension at 72 °C for 15 min. A nested PCR was performed with the Rdh552F/Rdh625R primer set, using PCR products obtained with the DHL-F1/DHL-R1 primer set. PCR reactions were performed in a total volume of 30 µL containing 0.75 U of OneTaq DNA Polymerase (NEB), 1X OneTaq Standard Buffer (NEB), 0.2 mM dNTPs mixture (Invitrogen), 0.3 µM of forward and reverse primers and 1–10 ng of template DNA. The following PCR conditions were used: an initial denaturation at 94 °C for 30 sec; [94 °C for 30 sec, 52 °C for 30 sec, 68 °C for 30 sec] X 25 cycles; a final extension at 68 °C for 7 min. Nested PCR products were cloned and inserted in the pCR 2.1- TOPO vector and *Escherichia coli* Oneshot TOP10 chemically competent cells were transformed with the TOPO^®^ TA Cloning Kit (Invitrogen) according to the manufacturer’s instructions. Recombinant plasmids from clones were extracted using the GeneElute™ Plasmid Miniprep Kit (SIGMA-ALDRICH^®^). Inserts were amplified with the M13f/M13r primer set (Supplementary Material 2) and sequenced via Macrogen Service (Amsterdam, The Netherlands). Nucleotide sequences obtained were compared to those in the NCBI GenBank database by the nucleotide-BLAST program, using Mega BLAST algorithm (“Highly similar sequences”).

PCR amplification of aerobic dechlorination genes was performed with degenerate or specific primers (Supplementary Material 2) designed on the sequences of the *dhlA* gene coding for a 1,2-DCA haloalkane dehalogenase, using the DNA extracted from aerobic OX microcosms as template. PCR reactions with the DHMf/DHMr and DHLA380F/548R primer sets were performed in a total volume of 20 µL containing 0.5 U of OneTaq DNA Polymerase (NEB), 1X OneTaq Standard Buffer (NEB), 0.2 mM dNTPs mixture (Invitrogen), 2 µM of DHMf/DHMr primers or 0.5 µM of DHLA380F/548R primers and 1–10 ng of template DNA. The following PCR conditions were used: an initial denaturation at 94 °C for 30 sec; [94 °C for 30 sec, 60 °C for 1 min, 68 °C for 30 sec] X 30 cycles; a final extension at 68 °C for 5 min. PCR protocol was optimized for PCR reactions with DHLA319F/603R primer set: reactions were performed in a total volume of total of 20 µL containing 0.5 U of OneTaq DNA Polymerase (NEB), 1X OneTaq GC Buffer, 0.2 mM dNTPs mixture (Invitrogen), 0.5 µM of forward and reverse primers and 1–10 ng of template DNA. The optimized PCR conditions were as follows: an initial denaturation at 94 °C for 30 sec; [94 °C for 15 sec, 65 °C for 15 sec, 68 °C for 20 sec] X 25 cycles; [94 °C for 15 sec, 60 °C for 20 sec, 68 °C for 20 sec] X 7 cycles; a final extension at 68 °C for 5 min. PCR products were sequenced and nucleotide sequences were analyzed as previously described.

### Statistical analysis

Differences in extracted DNA and diversity (number of OTUs from ARISA) between samples extracted from different filters (SFCA and PES membranes) were assessed by Wilcoxon rank test for non-normally distributed data (after Shapiro-Wilk test for normality). The correlation between the abundance of bacterial families (> 1% relative abundance) and the concentrations of the main CAHs in the site was determined by calculating the Spearman correlation coefficients.

## Results

### Physico-chemical parameters of the groundwater samples

The aquifer is characterized by a negative ORP (ranging from -51 to -462) and DO concentrations lower than 1 mg/L. The pH ranges from 5.99 to 7 and the average temperature is 23 °C (Supplementary Material 3 reports the physical and chemical parameters of the groundwater samples). The aquifer is contaminated by a variety of chlorinated solvents. 1,2-DCA and VC are the major contaminants, and their concentrations span six orders of magnitude (0.6–320000 µg/L for 1,2-DCA and 0.14–21000 µg/L for VC) with the highest levels detected in GW withdrawn from MW-D, followed by GW from MW-G and MW-E. The values registered in these GW samples far exceed the maximum concentration allowed by Italian law (3 µg/L for 1,2-DCA and 0.5 µg/L for VC, D. Lgs. 152/2006) (Supplementary Material 1 reports the concentration of chlorinated aliphatic hydrocarbons in the groundwater samples). Among the other CAHs measured in GW samples, 1,1-dichloroethylene, 1,2-dichloroethylene, 1,1-dichloroethane, 1,1,2-trichloroethane were also detected (Supplementary Material 1). Inorganic substances (sulfates, ammonium, boron) and metals (arsenic, iron, manganese, mercury, nickel) were also detected in the aquifer at concentrations higher than the law limits in some cases (Supplementary Material 3). MW-D monitoring for three consecutive years showed that the three main CAHs, namely 1,2-dichloroethane, vinyl chloride, and 1,1-dichloroethylene were reduced from 6-fold to 12-fold suggesting that natural attenuation could occur. With respect to the uncontaminated control sample from MW-K, the presence of CAHs was not detected (Supplementary Material 1), while the characteristics of groundwater (ORP, DO, temperature, pH) were similar to the rest of the aquifer (Supplementary Material 3).

### Groundwater microbial biomass

The microbial biomass in groundwater was estimated using total metagenomic dsDNA extracted from 1 L of filtered GW as a proxy [30]. GW samples were preliminarily filtered through two different systems, using 0.2 μm-pore-sized SFCA or PES filtering membranes. For each sample, the effect of the different filtration system on the amount of collected biomass and the relative extracted dsDNA was not significant (Wilcoxon V = 13, *p* = 0.55 data not shown). The amount of metagenomic dsDNA reported for SFCA filters varied across samples ranging from 0.247 ± 0.037 to 7.752 ± 0.327 ng/mL. For GW sampled from MW-D, the amount of dsDNA also changed over time, increasing significantly across the sampling events in three consecutive years (Table 2).

**Table 2:** Microbial biomass, 16S rRNA gene Illumina sequencing output and diversity indices of groundwater samples

	MW-K	MW-A	MW-B	MW-C	MW-D*			MW-E	MW-F	MW-G	MW-H
					1^st^	2^nd^	3^rd^				
**dsDNA** (ng/mL GW)	1.010 ± 0.131	0.329 ± 0.045	0.247 ± 0.037	0.28 ± 0.039	0.255 ± 0.044	1.766 ± 230	3.391 ± 0.461	0.398 ± 0.052	0.689 ± 0.125	7.752 ± 0.327	5.986 ± 0.838
**non-chimeric reads** (n)	51,070	40,041	40,025	58,214	51,563	60,204	36,384	46,058	47,041	44,089	45,084
**OTUs** (99% id.)	259	348	777	782	811	401	223	759	1888	126	361
**Bacteria** relative abundance (%)	98.30	99.55	92.8	91.39	94.18	99.69	99.84	87.07	84.9	99.93	95.29
**Archaea** relative abundance (%)	1.70	0.32	6.45	8.37	5.64	0.13	0.14	12.72	14.37	0.06	1.50
**Shannon** (H)	2.61	4.64	4.20	4.54	5.16	3.65	2.60	5.15	6.38	1.30	3.85
**Dominance** (D)	0.25	0.02	0.07	0.05	0.02	0.06	0.20	0.02	0.01	0.46	0.07
**Simpson** (1-D)	0.75	0.98	0.93	0.95	0.98	0.94	0.80	0.98	0.99	0.54	0.93

Microbial biomass was estimated as total dsDNA per volume of filtered groundwater for each sampling event (each value is the mean ± SE of total dsDNA extracted from two 0.2 μm-pore-sized SFCA filtering membranes). *For MW-D, 1^st^, 2^nd^, 3^rd^ are referred to the sampling events in three consecutive years

### High beta diversity among groundwater samples from different MWs

Automated ribosomal intergenic spacer analysis (ARISA) was used as a preliminary method to investigate the diversity among the microbial communities of GW sampled from the eight contaminated MWs. The cladogram built on ARISA profiles, shown in the Supplementary Material 4, revealed unexpected beta diversity among GW samples. The samples filtered through SFCA membranes showed a higher number of OTUs, in respect to PES (Wilcoxon V = 21, *p* = 0.035, data not shown) but both profiles clustered similarly (Supplementary Material 4). The microbial communities from the highest 1,2-DCA/VC-contaminated samples, i.e. MW-A, MW-D, MW-E and MW-G GW samples, clustered together. As the two filtering systems gave similar results, SFCA membranes were selected for the subsequent filtrations as they allowed to detect a higher richness.

### Spatial and temporal dynamics of groundwater microbial communities

**Fig. 2:**
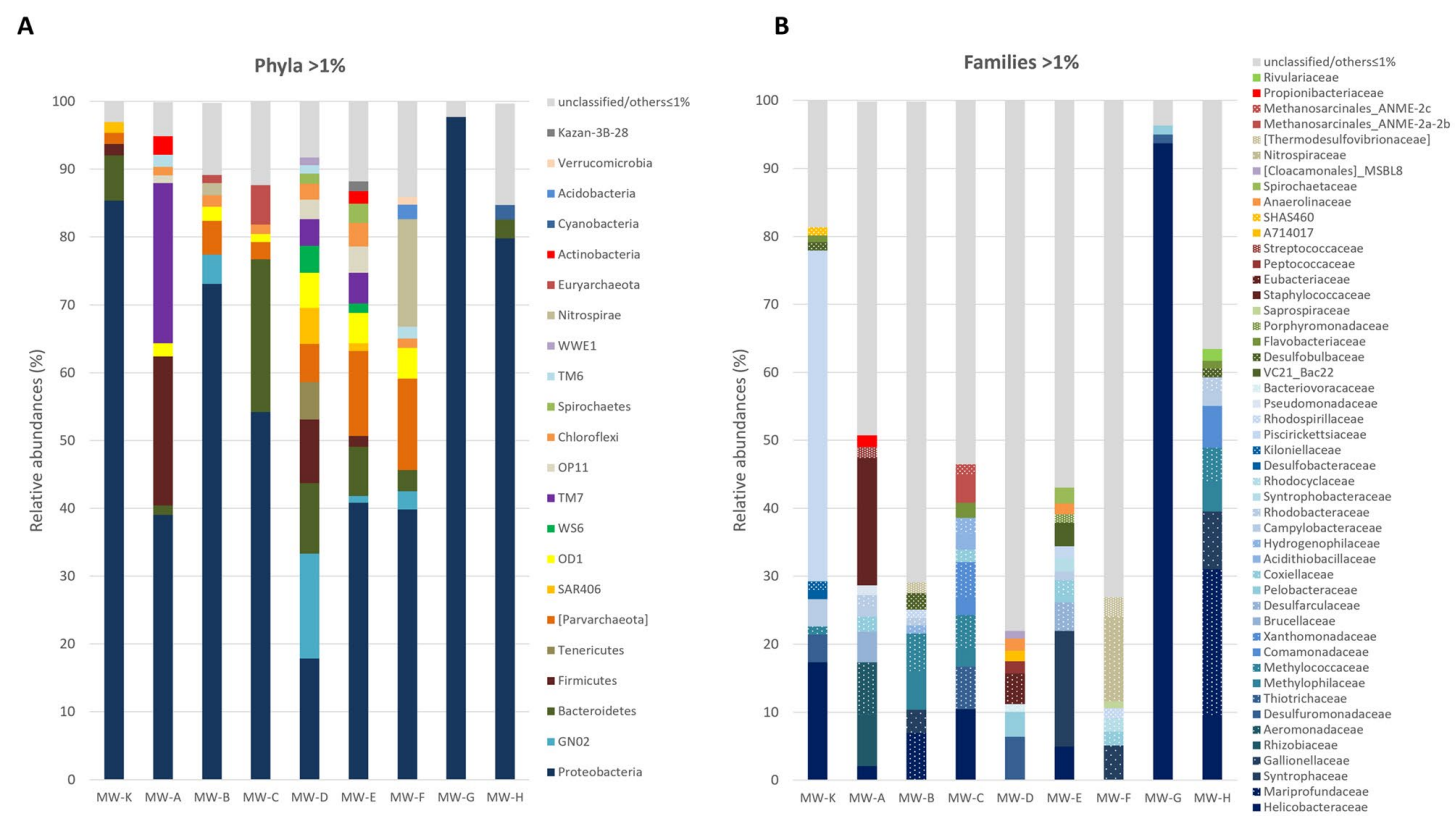
Groundwater microbiota. Bacterial and archaeal composition of groundwater sampled from the contaminated aquifer (MW-A to MW-H) and from an uncontaminated upstream well (MW-K) in the first sampling campaign at (**A**) phylum and (**B**) family level. Only taxa with relative abundance > 1% are shown. Unclassified/others includes unclassified taxa and identified taxa ≤ 1%

The 16S rRNA gene sequencing output is summarized in Table 2. The number of final reads for each GW sample, after the trimming, denoising and filtering processes, was between 40,025 (MW-B) and 60,204 (MW-D year 2); the number of OTUs, clustered at 99% identity, fell within a range from 126 (MW-G) to 1,888 (MW-F) (Table 2). The metagenomic analysis highlighted a high microbial diversity in the aquifer, with a total of 70 phyla, 223 families and 275 genera identified in all the samples; Bacteria were preponderant over Archaea with abundances ranging from 85 to 99.93% (Table 2). Among the 22 phyla with a relative abundance greater than 1% (Fig. 2A), Proteobacteria were the most abundant in all samples (from 18 to 98%), with MW-G almost exclusively represented by members of Proteobacteria. The most contaminated samples, MW-D and MW-E showed the highest number of phyla identified with abundance greater than 1%, including Proteobacteria (18-41%), Bacteroidetes (10%-7%), GN02 (16%-1%), Firmicutes (9%-2%) and, among the Archaea, Parvarchaeota (6-13%). From 4 up to 78% reads could not be assigned at the family level and the upper level of unassigned reads corresponded to the most contaminated sample MW-D. Among the identified bacterial families (Fig. 2B) at abundances > 1%, Helicobacteraceae were detected in 6 out of 9 MWs (2–94%), Methylococcaceae, Campylobacteraceae, Coxiellaceae were shared by 4 MWs at abundances of 1.2-4%. Gallionellaceae, Methylophilaceae and Desulfuromonadaceae were detected in 3 samples (1.3–8.5%). Piscirickettsiaceae were the most abundant taxon in the uncontaminated control MW-K (50.4%). The most abundant families that include dehalogenating taxa (Table 3) were Methylophilaceae, Methylococcaceae, Desulfuromonadaceae (average abundance 3–4%), Syntrophaceae, Brucellaceae, Eubacteriaceae, Comamonadaceae, Xanthomonadaceae, Dehalococcoidaceae, Peptococcaceae (1–2%). In particular, the OHRB genera *Dehalogenimonas* (0.1–1.4%), *Desulfomonile* (0.1–0.3%), and *Desulfuromonas* (0.2%) were detected within the families Dehalococcoidaceae, Syntrophaceae, and Desulfuromonadaceae, respectively. Among the identified genera involved in DAO metabolism there were *Ochrobactrum* (Brucellaceae) and *Stenotrophomonas* (Xanthomonadaceae), 4.5% and 0.5%, respectively, in MW-A. *Methylomonas* (Methylococcaceae, up to 3.8% in MW-C) and *Hydrogenophaga* (Comamonadaceae, 0.1–1.1%), were among the detected genera involved in cometabolic oxidation of CAHs (Table 3).

**Table 3:** Groundwater microbiota

						Relative abundance (%)							
Phylum	Family	Genus	Dehalogenating metabolism	Ref.	**MW-K**	**MW-A**	**MW-B**	**MW-C**	MW-D*	**MW-E**	**MW-F**	**MW-G**	**MW-H**
Chloroflexi	Dehalococcoidaceae				0.14	nd	0.60	0.06	0.26–1.75	0.97	0.06	0.10	nd
		***Dehalococcoides***	RD	[36]	nd	nd	nd	nd	nd^ED^	nd	nd	nd	nd
		***Dehalogenimonas***	RD	[36]	0.1	nd	nd	nd	0.14-1.42^ C^	0.87	nd	0.10	nd
Firmicutes	Peptococcaceae				nd	nd	nd	0.01	0.02–1.80	0.60	0.03	0.08	nd
		***Dehalobacter***	RD	[36]	nd	nd	nd	nd	0-0.01^ C^	nd	nd	nd	nd
		***Desulfitobacterium***	RD	[36]	nd	nd	nd	nd	nd^ED^	nd	nd	nd	nd
		*Desulfosporosinus*			nd	nd	nd	nd	0.02–1.76	0.52	nd	0.08	nd
		*Pelotomaculum*			nd	nd	nd	nd	0-0.01	0.05	0.03	nd	nd
Proteobacteria (α subdivision)	Brucellaceae				nd	4.49	nd	0.10	nd	nd	nd	nd	nd
		***Ochrobactrum***	DAO	[13]	nd	4.49	nd	0.10	nd	nd	nd	nd	nd
Proteobacteria (β subdivision)	Comamonadaceae				nd	0.16	0.30	2.64	0-0.03	0.03	0.78	0.02	6.15
		*Acidovorax*			nd	nd	nd	nd	0-0.03	nd	0.02	nd	nd
		*Aquabacterium*			nd	nd	nd	nd	nd	nd	0.03	nd	0.11
		***Hydrogenophaga***	ACo	[37]	nd	nd	nd	0.66	nd	nd	0.1	0.02	1.12
		*Limnohabitans*			nd	nd	nd	0.07	nd	nd	nd	nd	nd
		*Methylibium*			nd	0.02	0.19	0.26	nd	nd	0.28	nd	nd
		*Rhodoferax*			nd	nd	0.11	nd	nd	nd	0.11	nd	nd
		*Rubrivivax*			nd	nd	nd	nd	nd	nd	0.08	nd	nd
		*Thiomonas*			nd	nd	nd	1.57	0-0.01	0.03	0.02	nd	nd
Proteobacteria (β subdivision)	Methylophilaceae				0.17	nd	5.71	2.63	nd	nd	nd	nd	4.46
		*Methylotenera*			nd	nd	nd	2.16	nd	nd	nd	nd	0.13
		***Methylophilus***	DAO	[38]	nd	nd	nd	nd	nd	nd	nd	nd	0.10
Proteobacteria (ɣ subdivision)	Eubacteriaceae				nd	nd	nd	nd	0.95–4.52	0.15	nd	0.17	nd
		***Acetobacterium***	CoRD	[10]	nd	nd	nd	nd	0-0.05	nd	nd	nd	nd
Proteobacteria (ɣ subdivision)	Methylococcaceae				1.2	nd	5.53	4.99	nd	nd	0.43	nd	4.91
		*Methylocaldum*			nd	nd	nd	nd	nd	nd	0.03	nd	nd
		*Methylomicrobium*			nd	nd	nd	nd	nd	nd	0.29	nd	nd
		***Methylomonas***	ACo	[39]	0.65	nd	2.35	3.83	nd	nd	0.07	nd	1.92
Proteobacteria (ɣ subdivision)	Pseudomonadaceae				0.11	1.42	0.04	nd	0.02–0.15	0.01	0.25	nd	0.07
		***Pseudomonas***	CoRD DAO, ACo	[38, 39]	0.11	1.04	nd	nd	0-0.15	nd	0.1	nd	0.07
Proteobacteria (ɣ subdivision)	Xanthomonadaceae				0.09	0.69	0.61	5.13	0-0.09	0.20	0.36	nd	0.30
		*Dyella*			nd	nd	nd	0.13	nd	nd	0.01	nd	nd
		*Dokdonella*			nd	0.14	nd	nd	nd	nd	nd	nd	nd
		*Luteimonas*			nd	nd	nd	nd	nd	0.03	nd	nd	nd
		*Pseudoxanthomonas*			nd	nd	nd	nd	nd	0.01	nd	nd	nd
		***Stenotrophomonas***	DAO	[13]	nd	0.47	0.01	nd	0-0.09	nd	nd	nd	nd
		*Thermomonas*			nd	0.04	nd	nd	nd	nd	nd	nd	nd
Proteobacteria (δ subdivision)	Desulfuromonadaceae				4.07	0.08	0.01	0.49	6.37–13.27	0.86	0.10	1.31	0.09
		***Desulfuromonas***	RD	[36]	nd	nd	nd	nd	0.04-0.23^ C^	nd	nd	nd	nd
		*Desulfuromusa*			nd	nd	nd	nd	0-0.58	nd	nd	nd	nd
Proteobacteria (δ subdivision)	Syntrophaceae				nd	nd	0.45	0.44	0.10–0.93	17.01	0.53	0.02	0.13
		*Desulfobacca*			nd	nd	0.40	0.16	0-0.08	15.68	0.53	0.02	nd
		***Desulfomonile***	RD	[36]	nd	nd	0.05	0.28	nd	0.08	nd	nd	0.12
Proteobacteria (ε subdivision)	Campylobacteraceae				4.01	1.40	nd	0.71	0.04–1.27	1.28	nd	0.28	2.10
		*Arcobacter*			3.29	1.40	nd	0.71	0.02–1.16	1.25	nd	0.28	2.02
		***Sulfurospirillum***	RD	[36]	0.72	nd	nd	nd	0-0.11	0.03	nd	nd	nd

Relative abundance of identified bacterial families (with an abundance > 1%) which include known genera capable of dehalogenating metabolisms of chlorinated solvents (in bold type), in particular direct reductive dechlorination (RD), cometabolic reductive dechlorination (CoRD), direct aerobic oxidation (DAO), or aerobic cometabolism (ACo); for completeness all the genera identified in each family are reported. *For MW-D the range of relative abundance referred to the sampling events in three consecutive years is reported. ^ED^: Exclusively detected by PCR probing with OHRB-specific 16S rRNA gene primers; ^C^: Confirmed by PCR probing with OHRB-specific 16S rRNA gene primers; nd: not detected. Supplementary Material 5 includes also families < 1%

The families Desulfuromonadaceae, Eubacteriaceae, and Pelobacteraceae that were abundant in the contaminated wells, were positively correlated (*p* < 0.05) with the concentrations of 1,2-DCA and the total carcinogenic CAHs (chloroform, VC, 1,2-DCA, 1,1-dichloroethylene) (Supplementary Material 1), while Peptococcaceae were positively correlated (*p* < 0.05) with 1,2-DCA concentration only (data not shown). We could not find any other correlation between microbial biomass, bacterial diversity and chemical-physical parameters.

**Fig. 3:**
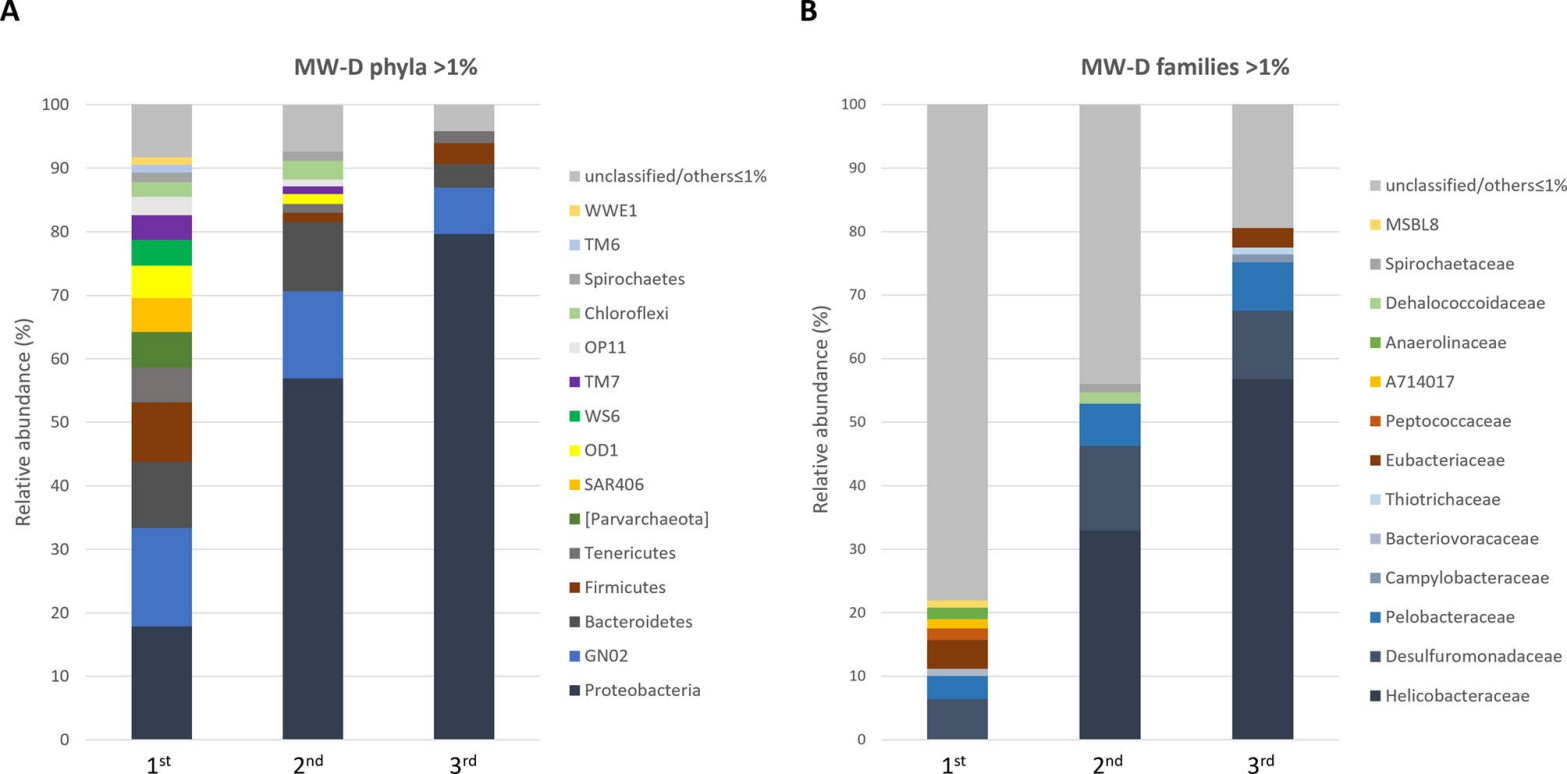
Groundwater microbiota of MW-D over time. Bacterial and archaeal community composition of groundwater samples from MW-D collected over three consecutive years (indicated as 1st, 2nd, 3rd ) at (**A**) phylum and (**B**) family level. Only taxa with relative abundance > 1% are shown. Unclassified/others includes unclassified taxa and identified taxa ≤ 1%

The microbiota of the most contaminated monitoring well, MW-D, was monitored for three consecutive years. While the microbial biomass increased over time, bacterial diversity decreased in terms of OTUs (approximately halving within each sampling), and Shannon index (Table 2). The histograms in Fig. 3 show phyla and families with relative abundance greater than 1%. At the phylum level, Proteobacteria was the dominant phylum whose abundance increased up to 80% in year 3. The abundance of GN02 division, Bacteroidetes and Firmicutes decreased from year 1 to year 3 by 2–3 fold. At the family level, Helicobacteraceae, Desulfuromonadaceae and Pelobacteraceae underwent a notable enrichment over time. Helicobacteraceae, in particular, reached an abundance of 57% in the third year and was dominated by the genera *Sulfuricurvum* and *Sulfurimonas*.

### Detection and abundance of potential dehalogenating taxa in GW

In order to retrieve the putative dechlorinating taxa, a literature survey was carried out considering all the mechanisms of direct reductive dechlorination (dehalorespiration, RD), cometabolic reductive dechlorination (CoRD), direct aerobic oxidation (DAO) and aerobic cometabolism (ACo). The families at abundance > 1% detected in the contaminated aquifer including known dehalogenating genera are shown in Table 3 (the complete data are shown in Supplementary Material 5) and include: Syntrophaceae, most abundant in MW-E (17%), but the most abundant genus detected, *Desulfobacca* is not known as dehalogenating, while the reductive dehalogenating *Desulfomonile* was detected at abundance < 1%; Desulfuromonadaceae, most abundant in MW-D (6.4 − 13.3%), including the genus *Desulfuromonas* (< 1%); Brucellaceae, almost exclusively in MW-A (4.5%), represented by the aerobic dehalogenating *Ochrobactum*; Methylococcaceae in MW-B, MW-C and MW-H (5-5.5%), represented mainly by *Methylomonas* known to oxidize CAH molecules cometabolically; Dehalococcoidaceae and Peptococcaceae mainly detected in MW-D (0.26–1.75% and 0-1.8%, respectively). These two families include the most renowned OHRB, namely *Dehalococcoides*,* Dehalogenimonas*,* Dehalobacter*, and *Desulfitobacterium*, but the presence of these key genera was not always assessed within the Illumina sequencing analysis.

In order to better detect the dehalogenating bacteria, in fact, a search for the known OHRB genera was carried out on the three MW-D DNA using a nested PCR approach based on 16S rRNA gene specific primers targeting each genus (*Dehalococcoides*,* Dehalogenimonas*,* Dehalobacter*,* Desulfitobacterium, Desulfuromonas*, Table 3). In particular, the 16S rRNA genes of *Desulfuromonas* and *Dehalogenimonas* were detected throughout the three years, in agreement with the results of 16S rRNA gene Illumina sequencing. Conversely, *Dehalococcoides* was exclusively detected by PCR as it was not identified by metagenomics. *Dehalobacter* was detected only in year 1 and *Desulfitobacterium* only in year 1 and 3; these genera were not identified by the metagenomic analysis, despite their family Peptococcaceae was detected with relative abundance > 1% in year 1. These latest results help to understand the dechlorination potential intrinsic to the MW-D sample and highlight the need to integrate the 16S rRNA metagenomic approach with genus specific search. Considering families that include aerobic dehalogenating genera, Xanthomonadaceae reached maximum abundance of 5% (in MW-E), and Pseudomonadaceae and Xanthobacteraceae were also detected at low levels (1.42 and 0.11%, respectively).

### Anaerobic dechlorinating microcosms

**Fig. 4:**
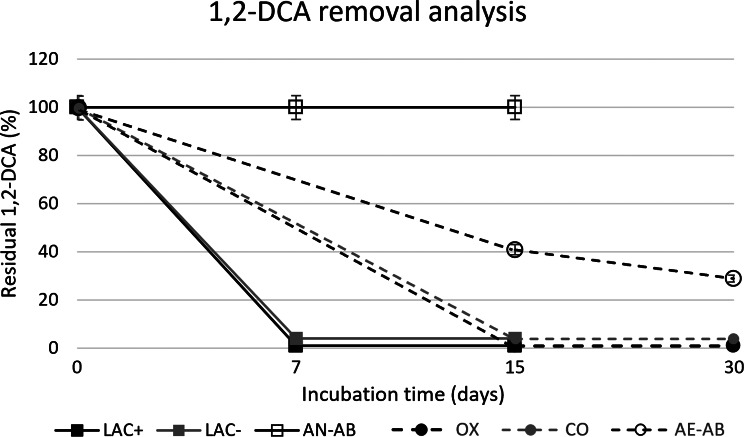
1,2-DCA removal analysis on anaerobic and aerobic microcosms. Anaerobic microcosms (solid line): LAC^+^ (black square), LAC^−^ (grey square) and AN-AB (empty square). Aerobic microcosms (dashed line): OX (black circle), CO (grey circle), AE-AB (empty circle)

GW sampled from the most contaminated MW-D at year 3 was used to set up anaerobic microcosms. Chemical monitoring of microcosms amended with (LAC^+^) and without (LAC^−^) sodium lactate revealed > 90% removal of 1,2-DCA after 7 days of incubation in both conditions, while no removal was observed in the abiotic control microcosms (the results of chemical monitoring are reported in the Fig. 4).

**Fig. 5:**
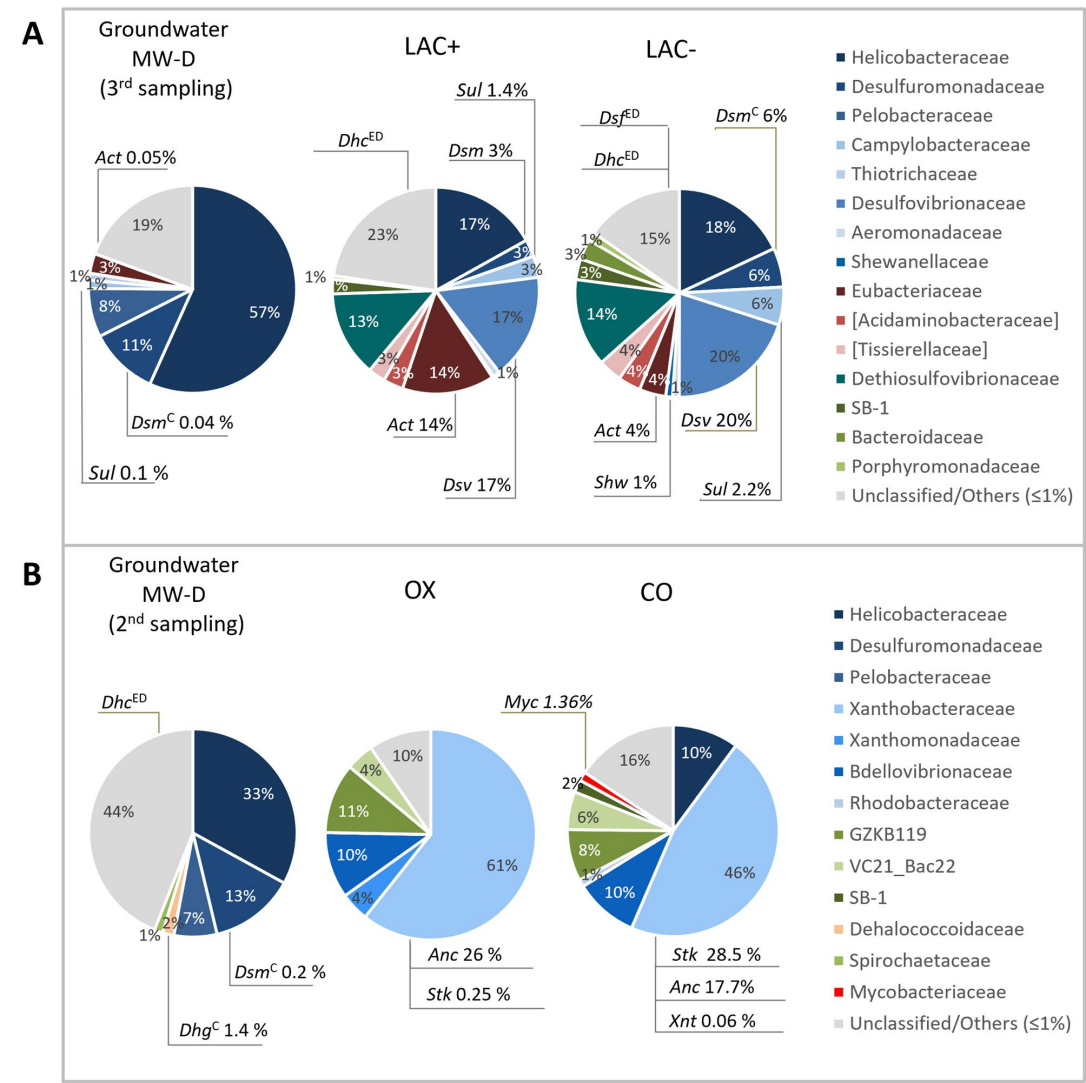
Microbiota of anaerobic and aerobic microcosms. Composition of bacterial communities of initial MW-D groundwater and after microcosm incubation under (**A**) anaerobic and (**B**) aerobic conditions. Microcosm conditions are reported in Table 1. Only families at abundance > 1% are shown. Unclassified/Others includes unclassified taxa and identified taxa ≤ 1%. Within each family only known dechlorinating genera are reported. ^ED^: exclusively detected by PCR on OHRB-specific 16S rRNA gene; ^C^: confirmed by PCR on OHRB-specific 16S rRNA gene. *Act: Acetobacterium; Anc: Ancylobacter; Dhc: Dehalococcoides; Dhg: Dehalogenimonas; Dsf: Desulfitobacterium; Dsv: Desulfovibrio; Dsm: Desulfuromonas; Myc: Mycobacterium; Shw: Shewanella; Stk: Starkeya; Sul: Sulfurospirillum; Xnt: Xanthobacter*

The bacterial biomasses of LAC^+^ and LAC^−^, estimated as extracted dsDNA (Table 4) were similar (124.5 ng/mL and 139.3 ng/mL, respectively) and much higher than GW (MW-D year 3, Table 2). The bacterial communities of LAC^+^ and LAC^−^ microcosms were analyzed by 16S rRNA gene Illumina sequencing and compared to MW-D year 3 (Fig. 5A). The diversity indices based on OTUs 99% (Table 4) showed that both microcosms were characterized by lower richness, higher diversity (Shannon) and lower dominance in respect to groundwater (Table 2), suggesting a shift of the microbial communities depending on the enrichment conditions imposed.

At the family level, both the LAC^+^ and LAC^−^ microcosms were dominated by Desulfovibrionaceae (Deltaproteobacteria), entirely composed of the sulfate-reducing genus *Desulfovibrio*, Helicobacteraceae (Epsilonproteobacteria) (17% and 18%), and Dethiosulfovibrionaceae (Synergistetes) (about 14%) (Fig. 5A). Eubacteriaceae, entirely represented by the fermenting genus *Acetobacterium* were detected in LAC^+^ (14.4%) and LAC^−^ (4%) microcosms. Desulfovibrionaceae, Dethiosulfovibrionaceae and Campylobacteraceae were enriched in microcosms in respect to groundwater. Conversely Helicobacteraceae decreased in respect to MW-D year 3. Eubacteriaceae (Firmicutes) were enriched only in LAC^+^ microcosm (14%), while the abundance observed in the LAC^−^ microcosm (4%) was similar to that in MW-D year 3 (3%). With respect to known dechlorinating genera, the genus *Sulfurospirillum* (Campylobacteraceae) was detected in both LAC^−^ and LAC^+^ microcosms (2% and 1.4%, respectively). The genus *Desulfuromonas* was enriched in microcosms (6% and 3% in LAC^−^ and LAC^+^, respectively) in respect to groundwater, although the family Desulfuromonadaceae was instead more abundant in groundwater (11%), but mainly made up of unclassified genera. The presence of *Desulfuromonas* in microcosms and in groundwater was also confirmed by probing with OHRB 16S rRNA gene specific primers. Nested PCR allowed the detection of *Dehalococcoides* in LAC^+^ and LAC^−^ microcosms and *Desulfitobacterium* in LAC^−^ microcosm, although they were not detected in the metagenome. Low abundances of the genus *Shewanella* (Shewanellaceae) were also detected (1% in LAC^−^ and < 0.5% in LAC^+^ microcosm).

**Table 4:** Microbial biomass, 16S rRNA gene Illumina sequencing output and diversity indices of the microcosms

	Anaerobic microcosms		Aerobic microcosms	
	LAC^−^	LAC^+^	OX	CO
**dsDNA** (ng/mL GW)	139.3	124.5	17.3	26.6
**non-chimeric reads** (n)	18,516	27,202	27,196	37,442
**OTUs** (99% id.)	119	79	89	155
**Shannon** (H)	3.64	3.03	2.20	2.79
**Dominance** (D)	0.04	0.08	0.20	0.14
**Simpson** (1-D)	0.96	0.92	0.80	0.86

Microcosms were set up from MW-D groundwater and incubated for 30 days. Microbial biomass was estimated as total dsDNA per volume of filtered microcosm. Microcosms conditions are described in Table 1. Microbial characterization of MW-D groundwater is reported in Table 2

### Detection of reductive dehalogenase genes in microcosms

The catabolic genes involved in reductive dehalogenation were detected in metagenomic DNA from LAC^+^ and LAC^−^ microcosms by PCR assays with degenerated and specific primers (Supplementary Material 2). A 300 bp amplicon, shorter than the expected size (400 bp), was obtained from the metagenomic DNA of both LAC^+^ and LAC^−^ microcosms with the DHL-F1/DHL-R1 primer set targeting a portion of the *dcaA* gene, encoding for a reductive dehalogenase specifically involved in 1,2-DCA dichloroelimination [4]. These amplicons were used as template in a nested PCR with the Rdh522F/Rdh625R primer set. The expected PCR products (95 bp) were cloned and 14 sequences (7 from LAC^+^ and 7 from LAC^−^ microcosm) presented 98–99% identity with the reductive dehalogenase genes *pceA* from *Desulfitobacterium* and *Dehalobacter*, both related to 1,2-DCA reductive dechlorination, and with a reductive dehalogenase gene cluster specifically active on 1,2-DCA (Table 5). An expected amplicon (326 bp) was also obtained from the metagenomic DNA from the LAC^−^ microcosm with the DHL-F2/DHL-R2 primer set, while no expected amplicons were obtained with primers targeting the *Shewanella* and *Sulfurospirillum rdhA* genes (data not shown), although both genera were detected by metagenomics.

**Table 5:** Detection and sequencing results of dehalogenase genes in microcosms

Microcosm name (n. of clones)	Target gene	Primer set	Sequence lenght	BLAST best match (nr/nt database)	Ident. (%)	Accession number	Reference
LAC^−^ microcosm (7 clones) LAC^+^ microcosm (7 clones)	*rdhA*	Rdh522F/Rdh625R	95 bp	Uncultured bacterium dichloroethane reductive dehalogenase gene cluster	98–99%	AM183919.1	[4]
				*Desulfitobacterium hafniense* Y51 orf0, orf1, pceA, pceB genes for transposase, hypothetical protein, PCE dehalogenase, PCE dehalogenase membrane-bound subunit, partial and complete cds	98–99%	AB070709.1	[40]
				*Dehalobacter restrictus* pce operon (pceABCT genes), strain PER-K23	98–99%	AJ439607.2	[41]
OX microcosm (2 clones)	*dhlA*	DHLA319F/DHLA 603R	156–173 bp	*Xanthobacter flavus* strain EL8 plasmid pDCA, complete sequence	100%	KU922121.1	[42]
				*Xanthobacter autotrophicus* strain EL4 plasmid pDCA	100%	KU922120.1	
				*Starkeya novella* strain EL1 plasmid pDCA, complete sequence	100%	KU922118.1	
				*Ancylobacter aquaticus* strain DH2 haloalkane dehalogenase (dhlA) gene, complete cds	100%	FJ573162.1	[43]
				*Xanthobacter flavus* clone UE-15 insertion sequence IS1247, haloalkane dehalogenase (dhlA), and transposase genes, complete cds	100%	AY561847.1	[44]
				*Xanthobacter autotrophicus* haloalkane dehalogenase (dhlA) gene, complete cds	100%	M26950.1	[45]

Microcosm composition is reported in Table 1

### Aerobic dechlorinating microcosms

GW sampled from the most contaminated MW-D at year 2 was used to set up aerobic microcosms biostimulated with air (OX) and a mixture of short chain HC (CO) to support aerobic dehalogenation and cometabolic oxidation, respectively. Chemical monitoring of the OX and CO microcosms showed > 90% removal of 1,2-DCA after 15 days of incubation; about 70% removal of 1,2-DCA was observed in abiotic control microcosms (Fig. 4).

The bacterial biomasses of aerobic microcosms, estimated as extracted dsDNA (Table 4), was 17.31 ng/mL and 26.59 ng/mL in OX and CO microcosms, respectively. The community composition was analyzed by 16S rRNA gene Illumina sequencing (Fig. 5B) and diversity indices were calculated (Table 4). Compared with groundwater, microcosms showed higher biomass, lower richness and lower Shannon index (H) values, suggesting the enrichment of specialized taxa in microcosms. Higher dominance (D) values reported for microcosm compared to groundwater suggested that fewer taxa were present in microcosms, and some of them, likely playing a major role within the community, were dominant over others.

The bacterial communities from the two aerobic microcosms showed similar composition, that was significantly different from GW (Fig. 5B). The family Xanthobacteraceae (Alphaproteobacteria) was strongly enriched in respect to GW and dominant in both microcosms (61% and 46% in OX and CO respectively). Within Xanthobacteraceae, the genera *Ancylobacter* (26% and 18% in OX and CO microcosms) and *Starkeya* (34% and 29%), both detected and dominant in microcosms, include known aerobic 1,2-DCA degraders [15, 42]. The family Bdellovibrionaceae increased up to 10% in both microcosms, while Xanthomonadaceae increased in OX microcosm only (4%). A slight increase of the family Mycobacteriaceae, represented by the genus *Mycobacterium*, was reported in CO microcosm (1.36%). Other uncharacterized lineages GZKB119 (11% and 8%) and VC21_Bac22 (4% and 6%) were enriched in OX and CO microcosms and SB-1 (2%) in CO microcosm. All these families and uncharacterized lineages were < 0.5% or below the detection limit in MW-D groundwater. Conversely, the family Desulfuromonadaceae, which includes known OHRB, was more abundant in groundwater (13%) and strongly reduced in microcosms (< 0.5%).

### Detection of the haloalkane dehalogenase gene *dhlA* in microcosms

The detection of abundant Xanthobacteraceae in OX microcosm, particularly the genera *Starkeya* and *Ancylobacter*, that include known aerobic 1,2-DCA dechlorinating strains, suggested the occurrence of the DhlA-mediated hydrolytic pathway for 1,2-DCA degradation. This hypothesis was confirmed by PCR probing the DNA extracted from OX microcosm with degenerated and specific primer sets targeting a portion of the *dhlA* gene, that encodes for the haloalkane dehalogenase DhlA. The expected size amplicon (284 bp) obtained with the DHLA319F/603R primer set was cloned, and sequences of 2 clones showed 100% identity with the plasmid-borne *dhlA* gene from *Xanthobacter* sp., *Ancylobacter* sp. and *Starkeya* sp. (Table 5).

## Discussion

### Fluctuating chemical physical parameters are associated to high beta diversity

In this paper we reported the results of the first microbiological survey of an aquifer with long-term contamination by chlorinated solvents, with peaks of the main contaminant, 1,2-DCA, reaching 0.3 g/L. Similar or even higher 1,2-DCA and VC concentrations were reported in other industrial sites [25, 29, 46] with peak concentrations of 1–2 g/L recorded at depths of 17–21 m [47]. Our study highlighted a great chemical and microbiological heterogeneity of the water mass under examination, both in space and in time.

The water samples collected from eight different monitoring wells (MW-A to MW-H), and over a period of three years from the most contaminated one (MW-D), present notable differences both in the chemical-physical parameters and in the levels of contamination by chlorinated solvents (mainly 1,2-dichloroethane, vinyl chloride and 1,1-dichloroethylene) and other chemical-physical parameters (Ec, DO, sulfate, boron, iron and manganese).

This large variability depends on the hydrodynamic water flow in the contaminated area and the establishment of two main water masses characterized by higher contamination (MW-D, MW-G, MW-E) and lower contamination (MW-A, MW-B, MW-C, MW-F, MW-H). Furthermore, the probable presence of Dense Non Aqueous Phase Liquids (DNAPLs) could represent a continuous source of release of chlorinated solvents to the aqueous phase, contributing to the uneven distribution of contamination in the area. In fact, 1,2-DCA fluctuating concentrations may vary with depth, with a shallower plume of lower concentrations and a deeper, higher concentration plume, but contaminant migration can also occur along preferential horizontal pathways [48].

The fluctuating contamination levels and hydrodynamic water flow were associated to high beta diversity of the GW microbiota of the eight MWs. Such heterogeneity was detected preliminarily by a fingerprinting approach (ARISA) and then confirmed by 16S rRNA gene metagenomic analysis. Clustering of GW ARISA profiles suggests that the presence of high concentrations of chlorinated solvents selects microbial communities that are more similar to each other and different from those present in less contaminated waters; nevertheless, a clear relationship between contamination levels and the presence of the main taxa identified in the microbial communities was not evidenced.

Despite the profound contamination of the aquifer, the presence of the most renowned bacterial genera capable of degrading CAHs was quite limited. The identified dechlorinating bacterial taxa were not abundant; coherently with the low ORP of the aquifer, OHRB bacterial taxa were more abundant than aerobic bacteria capable of oxidizing CAHs, especially in the most contaminated water samples.

We detected at very low abundance obligate and facultative OHR bacterial genera reported to specifically dechlorinate 1,2-DCA, namely *Dehaloccocoides* [5, 46], *Dehalogenimonas* [7, 29], *Desulfitobacterium* [4, 29], *Dehalobacter* [6, 46] and *Geobacter* [8].

In the case of *Dehalococcoides*, *Dehalogenimonas*, *Desulfitobacterium* and *Dehalobacter*, their low abundance coincided also with low abundance of the families to which they belong. Dehalococcoidaceae (in the phylum Chloroflexi) and Peptococcaceae (phylum Firmicutes) were represented below 2% in all the samples. In other cases, high abundance of families including known dehalogenating genera such as Synthrophaceae and Desulfuromonadaceae did not match with the abundance of the expected dehalogenating genera, *Desulfomonile* and *Desulfuromonas* respectively, suggesting the existence of other yet unidentified genera.

Such unexpected low relative abundance of dehalogenating taxa was associated to high abundance of sulfate-reducing and iron-reducing bacteria, known to compete with OHRB for the electron donors necessary for anaerobic respiration, in CAHs contaminated water samples [49].

Natural attenuation in MW-D is associated to biomass increase, reduced bacterial diversity and enrichment of Helicobacteraceae, Desulfuromonadaceae and Pelobacteraceae.

MW-D resulted as the most contaminated well (mainly by 1,2-DCA, VC and 1,1-dichloroethylene) at the first sampling campaign. Thus, this MW was selected for chemical and microbiological monitoring for three years. Significant reduction of the three main contaminants (from 6-fold to 12-fold) was associated to increased bacterial biomass (10-fold) and decreased diversity, suggesting a specialization of the microbiota. However, the main shifts in MW-D bacterial communities did not correspond to an increase of known OHRB or other dehalogenating taxa. Conversely, we observed a sensitive increase of Proteobacteria, mainly represented by Helicobacteraceae including only two genera, *Sulfuricurvum* and *Sulfurimonas.* Helicobacteraceae does not include known dehalogenating bacteria although their coexistence in CAHs and HCs contaminated water has been reported [50]. Members of this family carry out oxidation of reduced sulfur compounds in chemolithotrophic metabolisms. *Sulfuricurvum* and *Sulfurimonas* in particular, are chemolithoautotrophic and anaerobic bacteria that can facultatively oxidize sulfide and hydrogen using either oxygen or nitrate as an electron acceptor thus providing energy for dehalogenation of chlorinated compounds in coastal sediments contaminated by PCB [51]. These two genera were found highly abundant and significantly enriched in groundwaters contaminated by CAHs and hydrocarbons revealing their ability to tolerate the contaminants [52]. *Sulfuricurvum* was enriched in the reductive compartment of a bioelectrochemical system (BES) fed by real contaminated groundwater, based on sequential reductive/oxidative dechlorination for perchloroethylene (PCE) removal [53]. The observed coexistence of the key chemolithotrophic oxidation of reduced sulfur compounds with the degradation of chlorinated compounds [54] suggests that the role of ε-proteobacteria in providing energy for decontamination could be more important than actually considered and worth to be clarified [51]. Conversely, the reports on the ability of *Sulfuricurvum kujiense* strain YK-3 to dechlorinate TCE under sulfate-reducing conditions seem inconsistent (see Tsai et al. [55] and references therein).

Pelobacteraceae do not include known dehalogenating genera but, interestingly, this family doubled during the natural attenuation monitoring period and its abundance was correlated to the concentration of 1,2-DCA and total carcinogenic CAHs. The role of Pelobacteraceae is not yet totally defined. The C_2_H_2_-fermenting *Pelobacter* SFB93 allows recovery of the dehalogenating activity of *D.* *mccartyi* strain 195, by removal of acetylene, that is an inhibitor of trichloroethene (TCE) degradation, that is generated by chemical degradation [56]. Moreover within Deltaproteobacteria, *Pelobacter* genomes share mobile elements and genomic features with the OHRB *Geobacter* SZ suggesting the possibility of lateral acquisition of DNA including the genomic island enabling to respire PCE and TCE [57].

Desulfuromonadaceae and Campylobacteraceae are the families including known reductive dehalogenating genera, that increase most during the monitoring period of the MW-D. Although not dominant in the microbial community, we suggest that members of these families (i.e. *Desulfuromonas* and *Sulfurospirillum*), supported by energy provided by chemolithotrophic metabolisms, could be actors of natural attenuation of 1,2-DCA and VC in the aquifer [58, 59]. Moreover, the high percentage of unclassified taxa supports the hypothesis that still unknown dechlorinating bacteria could be present in the studied water. The potential for the biodegradation of 1,2-DCA was better investigated with the microcosm studies.

### Microbial community shift during enrichment in microcosms

#### *Desulfovibrio*, *Desulfuromonas*, *Sulfurospirillum* and *Acetobacterium* are enriched in dehalogenating anaerobic microcosms

In anaerobic microcosms the degradation of 1,2-DCA was associated to the enrichment of *Desulfovibrio*,* Sulfurospirillum* and *Desulfuromonas.* These genera are known to include non-obligate dehalogenating strains, but none of them is reported as 1,2-DCA degrader. *Desulfovibrio* sp. is able to couple lactate or acetate oxidation to reductive dehalogenation of chlorophenol [60] and bromophenol compounds 61]. *Sulfurospirillum* is a facultative OHRB that mainly dechlorinates chlorinated ethenes by using different substrates, including lactate, as electron donors [59, 62]. The reductive dehalogenase PceA gene of the tetrachloroethene-degrading *Sulfurospirillum multivorans* was the first *rdhA* gene to be sequenced in 1998 [63]. *Desulfuromonas* has been reported to couple PCE reduction to the oxidation of acetate or lactate [58] and to cometabolically dechlorinate TCE in sulfate- or sulfur- reducing bacterial communities [64]. Most organohalide-respiring bacteria however, have been tested only on a reduced range of halogenated compounds and there is little or no correlation between phylogenetic affiliation and chlorinated substrate specificities [65]. At functional gene level, many reductive dehalogenase genes that have been sequenced so far have low sequence similarity and fewer than 1% of all the sequences have a characterized function [66]. This poor correlation between phylogenetic affiliation, *rdh* gene sequence similarity and substrate specificity, makes functional prediction from taxonomic and sequence information very difficult [65]. For this reason, the *rdhA* gene sequence of our anaerobic microcosm cannot be uniquely attributed to one of the genera detected. The closest homologs of our *rdhA* gene belong to the PCE dehalogenase operon of two genera *Desulfitobacterium* and *Dehalobacter*, that we did not detect in the metagenome, and to an uncultured 1,2-DCA dehalogenating bacterium. Assuming that PCE dehalogenases are effective in the dehalogenation of PCE and 1,2-DCA, we suggest that dehalogenation of 1,2-DCA in our anaerobic microcosms could be carried out by *Desulfovibrio* and *Desulfuromonas*, known as PCE degraders [36] and yet unknown as 1,2-DCA respirers.

Alternatively, *Desulfovibrio* may play a role in facilitating organohalide respiration in syntrophic associations with dechlorinating strains [67, 68]. In these associations, lactate fermentation carried out by *Desulfovibrio* and subsequent H_2_ production are able to sustain the growth of the dechlorinating strain in coculture [69]. However, we detected very low abundance of the obligate OHRB (*Dehalococcoidaceae*, *Dehalobacter*,* Desulfitobacterium*) to sustain this hypothesis. The conditions imposed in anaerobic microcosms were enough to enrich the bacterial community with RD Proteobacteria up to 30% abundance, but probably were not reductive enough to allow enrichment of obligate OHRB such as *Dehalococcoides*, that is highly susceptible to oxygen inhibition.

The addition of lactate in LAC^+^ microcosms did not cause drastic changes in the microbial composition in respect to the LAC^−^ microcosms but clearly stimulated *Acetobacterium*, a homoacetogenic fermenting genus [70] that is able to cometabolically dechlorinate 1,2-DCA to ethene with lactate as substrate, thanks to the ability of Co(I) corrinoids (vitamin B_12_) to reduce halogen substituted carbon atoms in halogenated compounds during acetogenesis [10]. Considering that also in the absence of lactate almost complete 1,2-DCA removal was obtained in 7 days, we conclude that lactate addition was not necessary for 1,2-DCA degradation, but lactate can shift 1,2-DCA degradation to cometabolic pathways. *Acetobacterium* degradation by anaerobic cometabolic pathway, however, is not usually exploited in bioremediation, as cometabolic reactions are usually slow and their contribution to the overall contaminant removal cannot be precisely accounted for, as they are fortuitous reactions mediated by nonspecific enzymes operating on other (growth) substrates.

#### Abundance of Xanthobacteraceae in dehalogenating aerobic microcosms

In aerobic microcosms, Xanthobacteraceae (Alphaproteobacteria) and Bdellovibrionaceae (Deltaproteobacteria) were strongly enriched in respect to GW, irrespective of the addition of the HCs mixture that aimed to enrich the cometabolic degraders. The dominant genera within both microcosms, *Ancylobacter* and *Starkeya*, are two Xanthobacteraceae already known to aerobically degrade 1,2-DCA [15, 42, 71] using the DhlA-mediated hydrolytic pathway [45]. Their abundance above 25% is clearly related to 1,2-DCA removal in the microcosms. Since the first isolation and characterization of *Ancylobacter aquaticus* and *Xanthobacter autotrophicus*, able to grow on 1,2- DCA [14, 15], only a few other aerobic strains capable of growing on 1,2-DCA as the sole carbon source have been isolated and assigned to the genera *Xanthobacter*,* Starkeya* and *Leifsonia* [42]. To date, the only known hydrolytic pathway operating in 1,2-DCA degrading bacteria is mediated by the DhlA enzyme, that is encoded by the *dhlA* gene, shared by all these bacteria (excluding *Leifsonia*, where no *dhlA* gene nor the enzyme was detected). This gene was originally discovered in the nitrogen-fixing hydrogen bacterium *X. autotrophicus* GJ10 [45] and later found identical in other *Xanthobacter*,* Ancylobacter* and *Starkeya* isolates with different geographical origins [15, 42, 44]. In these isolates, the *dhlA* gene is located on different plasmids (e.g. on a 225 kb linear plasmid for *X. autotrophicus* GJ10 [72] and on a 37 kb circular plasmid, named pDCA for strains isolated by Munro et al. [42]) and associated with insertion sequences and transposases, suggesting the high mobility of the genes, in a restricted range of genera, although the mechanisms of mobilization and distribution of this gene are not clear yet [12, 42]. The *dhla* sequences from our aerobic microcosms were also 100% identical to the plasmid borne *dhla* of *Xanthobacter*, *Starkeya* and *Ancylobacter*. Further characterization of the novel aerobic consortia obtained from our microcosms, and isolation of the detected degraders are in progress (Sciré Calabrisotto et al., unpublished).

## Conclusions

In this research we analyzed for the first time the bacterial microbiome of a CAHs-contaminated aquifer. A slow natural attenuation, monitored in situ for three years, was associated with an increase of Desulfuromonadaceae and Campylobacteraceae, that include putative dehalogenating genera but not known as 1,2-DCA degraders. Although novel dehalogenating taxa are likely to be discovered from environments endowed with organohalide-respiring activity [65], our approach, based on probes designed on known OHRB, limited the identification of the novel dehalogenating taxa in our study. Among known dehalogenating genera, we propose that the PCE degraders *Desulfovibrio* and *Desulfuromonas* [36] are also 1,2-DCA respirers, although confirmation by further studies is needed.

If the detection of novel anaerobic 1,2-DCA degraders seems still possible with increasing number of sites investigated, no novel taxa or even novel oxidative pathways have been reported since 2016 [42]. The detection of novel 1,2-DCA-degrading aerobic taxa seem to be limited within Xanthobacteraceae [15, 42, 44, 45] that rely on the haloalkane dehalogenase DhlA, currently the only known hydrolytic haloalkane dehalogenase active on 1,2-DCA.

Despite the negative ORP (mV) and DO < 0.4 mg/L recorded in the aquifer, we successfully obtained degradation of 1,2-DCA in both anaerobic and aerobic microcosms. Indeed the novelty of the manuscript relies on the simultaneous study of the two conditions, that are rarely tested in the same site. Under low oxygen conditions 1,2-DCA and VC degradation occurs and is positively correlated to the abundance of the *dhlA* [29]. Although anaerobic dehalorespiration is faster than aerobic dehalogenation [3], we propose that bioremediation strategy based on DAO with oxygen insufflation, that is free from toxic intermediates and final products, could be applied to the contaminated aquifer of the present study as an ecofriendly, efficient and cost-effective approach.

## Electronic supplementary material

Below is the link to the electronic supplementary material.

Supplementary Material 1

Supplementary Material 2

Supplementary Material 3

Supplementary Material 4

Supplementary Material 5

## Data Availability

The datasets generated during the current study have been deposited in the NCBI short reads archive (SRA) database under BioProject ID PRJNA1134153 (http://www.ncbi.nlm.nih.gov/bioproject/1134153).
