# Supplementary material for: 1,2-DCA biodegradation potential of an aquifer assessed in situ and in aerobic and anaerobic microcosms
Source: Environ Microbiome. 2024 Dec 18;19:106. doi: 10.1186/s40793-024-00650-w (PMC11658234; doi:10.1186/s40793-024-00650-w)
Supplement: Supplementary file 1 — Supplementary Material 1 [file 40793_2024_650_MOESM1_ESM.docx]

**Supplementary Material 1.** Concentration of chlorinated aliphatic hydrocarbons (CAHs) in the (GW) groundwater samples.

|  | **MW-K** | **MW-A** | **MW-B** | **MW-C** | **MW-D*** | | | **MW-E** | **MW-F** | **MW-G** | **MW-H** |
| --- | --- | --- | --- | --- | --- | --- | --- | --- | --- | --- | --- |
|  |  |  |  |  | 1^st^ | 2^nd^ | 3^rd^ |  |  |  |  |
| Chloroform (µg/L) | <0.010 | <1.3 | <0.013 | <0.013 | <13 | <13 | <1.30 | **16** | <0.013 | <13 | <0.013 |
| Vinyl chloride (µg/L) | <0.050 | **11000** | **4.8** | 0.14 | **21000** | **12000** | **3200** | **1600** | **0.59** | **8300** | **5** |
| 1,2-Dichloroethane (µg/L) | 0.067 | <4.7 | 1.7 | 0.6 | **220000** | **320000** | **35000** | **25000** | 1.9 | **130000** | **16** |
| 1,1-Dichloroethane (µg/L) | 0.021 | 33 | 23 | 1 | 160 | 84 | 42 | 130 | 0.93 | 53 | 3.1 |
| 1,1-Dichloroethylene (µg/L) | <0.005 | **48** | **0.23** | **0.39** | **1200** | **500** | **94** | **72** | **0.33** | **180** | **0.9** |
| 1,2-Dichloroethylene (µg/L) | <0.10 | 17 | 0.92 | 0.17 | **120** | <84 | 29 | 6.9 | 5.8 | <39 | 3.3 |
| 1,1,2-Trichloroethane (µg/L) | <0.010 | <1.7 | <0.017 | <0.017 | **78** | **65** | **2.2** | **17** | <0.017 | <17 | <0.017 |

Values that exceed the minimum concentration required by Italian law (D.Lgs. 152/2006) are highlighted with bold type. *For MW-D, 1^st^, 2^nd^, 3^rd^ are referred to the sampling events in three consecutive years. MW-K is the uncontaminated GW sample.
