# Supplementary material for: 1,2-DCA biodegradation potential of an aquifer assessed in situ and in aerobic and anaerobic microcosms
Source: Environ Microbiome. 2024 Dec 18;19:106. doi: 10.1186/s40793-024-00650-w (PMC11658234; doi:10.1186/s40793-024-00650-w)
Supplement: Supplementary file 2 — Supplementary Material 2 [file 40793_2024_650_MOESM2_ESM.docx]

**Supplementary Material 2.** Primers used in this study.

| Primers set | Target | Primer sequence (5’🡪3’)* | Orientation | Annealing temp (°C) | Product size (bp) | Reference |
| --- | --- | --- | --- | --- | --- | --- |
| 27F  1492R | 16S rRNA gene (universal primers) | AGAGTTTGATCMTGGCTCAG  TACGGYTACCTTGTTACGACTT | Forward  Reverse | 50 | 1500 | [1] |
| M13f  M13r | pCR™4-TOPO® plasmid insert | GTAAAACGACGGCCAG  CAGGAAACAGCTATGAC | Forward  Reverse | 55 | Insert size | - |
| Dhc1f  Dhc264r | *Dehalococcoides* 16S rRNA gene | GATGAACGCTAGCGGCG  CCTCTCAGACCAGCTACCGATCGAA | Forward  Reverse | 59 | 264 | [2] |
| BL-DC-142f  BL-DC-1351r | *Dehalogenimonas* 16S rRNA gene | GTGGGGGATAACACTTCGAAAGAAGTGC  AACGCGCTATGCTGACACGCGT | Forward  Reverse | 63 | 1199 | [3] |
| Dhb477f  Dhb647r | *Dehalobacter*  16S rRNA gene | GATTGACGGTACCTAACGAGG  TACAGTTTCCAATGCTTTACGG | Forward  Reverse | 63 | 170 | [2] |
| Desulfo16sF3  Desulfo16sR5 | *Desulfitobacterium*16S rRNA gene | TTARTAGATGGATCCGCGTCTG  TTTCCGATGCAGTCCCAGG | Forward  Reverse | 51 | 500 | [4] |
| Dsm16Sf  Dsm16Sr | *Desulfuromonas* 16S rRNA gene | AACCTTCGGGTCCTACTGTC  GCCGAACTGACCCCTATGTT | Forward  Reverse | 58 | 835 | [5] |
| DHL-F1  DHL-R1 | *dcaA* gene | GGACCTCGTTGGACTCC  GGCAAATCCCATGGCATTA | Forward  Reverse | 54 | 400 | [6] |
| DHL-F2  DHL-R2 | *dcaA* gene | GTTAAAAAGGCAGCCTGTT  GTAAACTTTCCCCGTCGC | Forward  Reverse | 54 | 326 | [6] |
| PceAFor1  DcaBRev | *dcaA* gene | ACGTGCAATTATTATTAAGG  TGGTATTCACGCTCCGA | Forward  Reverse | 54 | 1944 | [6] |
| Rdh522F  Rdh625R | *dcaA* gene | ATGACCAATGAAATAGCTAATGA  TGTTGGCCTTGTTGTCGCACACT | Forward  Reverse | 52 | 95 | [7] |
| RDH_Group20F  RDH_Group20R | *Sulfurospirillum rdhA* gene | GCCTGAACTCTCAAGAAGAG  GCTTACAGTGTCTCTAAAATGGTC | Forward  Reverse | 50 | 1470 | [8] |
| Sul-rdhA-f  Sul-rdhA-r | *Sulfurospirillum rdhA* gene | TTRGTRGGTRTTGCAAGATT  CTTGTCCTAAACCTGCTTC | Forward  Reverse | 53 | 394 | [9] |
| RDH_group31BF  RDH_Group31R | *Shewanella*  *rdhA* gene | TGGATAAAACAMARAGTYGAAGG  GRCAWGMHTCRGCRCA | Forward  Reverse | 50 | 960 | [8] |
| ceRD2L  RD7 | *rdh* gene | GCAGCACGCCTTTTTGGIGCIKMIYTNGTIGG  AANGGRCAIACIGCIWCRCA | Forward  Reverse | 47 | 700-900 | [10] |
| ceRD2S  RD7 | *rdh* gene | GCAGCACGCCTTTTTGGIGCIKMIWSIGTIGG  AANGGRCAIACIGCIWCRCA | Forward  Reverse | 47 | 700-900 | [10] |
| DHMf  DHMr | *dhlA* gene | GGCGAGCCCACCTGGAGYTAC  GWMKYGTCRGGGAARGGCGC | Forward  Reverse | 60 | 450 | [11] |
| DHLA 380F  DHLA 548R | *dhlA* gene | GATTTTTGGGGCTGACCTTA  GATGGCGTAACCAGATCGTA | Forward  Reverse | 60 | 168 | [12] |
| DHLA 319F  DHLA 603R | *dhlA* gene | CTTGCACTAATCGAACGGCTTG  AGCTTCGGTCAGTGTGGGCG | Forward  Reverse | 65 | 284 | [12] |

*Abbreviations of degenerate nucleotides: I, inosine; K, G/T; M, A/C; N, A/C/G/T; R, A/G; S, C/G; W, A/T; Y, C/T; H, ACT.

1. Frank, J. A., Reich, C. I., Sharma, S., Weisbaum, J. S., Wilson, B. A., & Olsen, G. J. (2008). Critical evaluation of two primers commonly used for amplification of bacterial 16S rRNA genes. Applied and environmental microbiology, 74(8), 2461-2470.
2. Grostern, A., & Edwards, E. A. (2006). Growth of Dehalobacter and Dehalococcoides spp. during degradation of chlorinated ethanes. *Applied and* Environmental Microbiology, 72(1), 428-436.
3. Chen, J., Bowman, K. S., Rainey, F. A., & Moe, W. M. (2014). Reassessment of PCR primers targeting 16S rRNA genes of the organohalide-respiring genus *Dehalogenimonas*. Biodegradation, 25(5), 747-756.
4. Marzorati, M., Borin, S., Brusetti, L., Daffonchio, D., Marsilli, C., Carpani, G., & de Ferra, F. (2006). Response of 1, 2-dichloroethane-adapted microbial communities to ex-situ biostimulation of polluted groundwater. *Biodegradation*, *17*(2), 41-56.
5. Löffler, F. E., Sun, Q., Li, J., & Tiedje, J. M. (2000). 16S rRNA gene-based detection of tetrachloroethene-dechlorinating *Desulfuromonas* and *Dehalococcoides* species. Applied and Environmental Microbiology, 66(4), 1369-1374.
6. Marzorati, M., De Ferra, F., Van Raemdonck, H., Borin, S., Allifranchini, E., Carpani, G., ... & Daffonchio, D. (2007). A novel reductive dehalogenase, identified in a contaminated groundwater enrichment culture and in *Desulfitobacterium dichloroeliminans* strain DCA1, is linked to dehalogenation of 1, 2-dichloroethane. Applied and environmental microbiology, 73(9), 2990-2999.
7. Carpani, G., Marchesi, M., Pietrini, I., Alberti, L., Zaninetta, L. M., Shouakar-Stash, O., & de Ferra, F. (2021). 1, 2-DCA Natural Attenuation Evaluation in Groundwater: Insight by Dual Isotope 13C/37Cl and Molecular Analysis Approach. Water, 13(5), 728.
8. Hug, L. A., & Edwards, E. A. (2013). Diversity of reductive dehalogenase genes from environmental samples and enrichment cultures identified with degenerate primer PCR screens. Frontiers in microbiology, 4, 341.
9. Buttet, G. F., Holliger, C., & Maillard, J. (2013). Functional genotyping of *Sulfurospirillum* spp. in mixed cultures allowed the identification of a new tetrachloroethene reductive dehalogenase. Applied and environmental microbiology, 79(22), 6941-6947.
10. Regeard, C., Maillard, J., & Holliger, C. (2004). Development of degenerate and specific PCR primers for the detection and isolation of known and putative chloroethene reductive dehalogenase genes. Journal of Microbiological Methods, 56(1), 107-118.
11. Govender, A., & Pillay, B. (2011). Characterization of 1, 2-dichloroethane (DCA) degrading bacteria isolated from South African wastewater. African Journal of Biotechnology, 10(55), 11567-11573.
12. Munro, J. E., Liew, E. F. & Coleman, N. V. (2013). Adaptation of a membrane bioreactor to 1,2-dichloroethane revealed by 16S rDNA pyrosequencing and *dhlA* qPCR. Environ Sci Technol 47, 13668–13676.
