# Supplementary material for: 1,2-DCA biodegradation potential of an aquifer assessed in situ and in aerobic and anaerobic microcosms
Source: Environ Microbiome. 2024 Dec 18;19:106. doi: 10.1186/s40793-024-00650-w (PMC11658234; doi:10.1186/s40793-024-00650-w)
Supplement: Supplementary file 3 — Supplementary Material 3 [file 40793_2024_650_MOESM3_ESM.docx]

**Supplementary Material 3.** Physical and chemical parameters of the groundwater samples.

|  | **MW-K** | **MW-A** | **MW-B** | **MW-C** | **MW-D*** | | | **MW-E** | **MW-F** | **MW-G** | **MW-H** |
| --- | --- | --- | --- | --- | --- | --- | --- | --- | --- | --- | --- |
|  |  |  |  |  | 1^st^ | 2^nd^ | 3^rd^ |  |  |  |  |
| ORP (mV) | -99 | -326 | -92 | -389 | -198 | -243 | -199 | -462 | -247 | -228 | -51 |
| DO (mg/L) | 0.25 | 0.04 | 0.16 | 0.04 | 0.32 | 0.13 | 0.27 | - | 0.05 | 0.35 | 0.91 |
| Temperature (℃) | 20.7 | 21.86 | 22.53 | 21.28 | 21.92 | 30.51 | 23.3 | 21.45 | 22.48 | 23.71 | 22.61 |
| pH | 6.8 | 6.42 | 6.62 | 6.96 | 5.99 | 6.37 | 6.1 | 6.3 | 7 | 6.37 | 6.77 |
| EC (µS/cm) | 35570 | 8215 | 13877 | 11372 | 22404 | 19620 | 19532 | 10422 | 8052 | 23467 | 21944 |
| Total dissolved solids (mg/L) | 30000 | 5700 | 8100 | 8200 | 14000 | 13000 | - | 7500 | 5500 | 14000 | 14000 |
| Chloride (mg/L) | 17200 | 1600 | 3800 | 2700 | 7900 | 5300 | - | 1800 | 2100 | 7300 | 7100 |
| Fluoride (µg/L) | 490 | 1000 | 1400 | 1000 | 670 | 720 | - | 910 | 1300 | 880 | 1000 |
| Sulfate (mg/L) | **1630** | **1600** | **2400** | **2700** | **1700** | **1600** | - | **1300** | **1000** | **2500** | **1900** |
| Phosphate(mg/L) | 0.63 | 0.06 | 0.11 | 0.15 | 0.97 | <0.06 | - | 0.06 | 0.1 | 0.16 | 0.16 |
| Ammonium (mg/L) | **17.6** | **6.9** | **350** | **3.4** | **100** | **120** | - | **1.8** | **22** | **370** | **220** |
| Aluminum (µg/L) | <100 | 13 | <11 | 18 | <11 | <28 | - | 27 | 16 | 18 | 13 |
| Arsenic (µg/L) | **25.3** | **18** | 4 | <1.8 | 7.2 | 4.8 | - | **43** | <1.8 | 9.8 | 4.7 |
| Boron (µg/L) | **4540** | **1200** | **1900** | **3500** | **3600** | **3800** | - | **4200** | **1200** | **2800** | **2900** |
| Cobalt (µg/L) | 10.2 | <2.1 | 3.9 | <2.1 | <2.1 | 0.56 | - | 2.4 | <2.1 | <2.1 | <2.1 |
| Iron (µg/L) | **37900** | **1700** | **4400** | 110 | **7300** | **5100** | - | **36000** | **2000** | **4800** | **7100** |
| Manganese (µg/L) | **11200** | **2200** | **890** | **2100** | **2600** | **2100** | - | **2000** | **440** | **2600** | **1300** |
| Mercury (µg/L) | <5.0 | <0.49 | <0.49 | <0.49 | <0.49 | <0.42 | - | <0.49 | <0.49 | **1.2** | <0.49 |
| Nickel (µg/L) | <5.0 | 3.5 | 5 | <2.2 | 4.3 | 6.1 | - | <2.2 | 8.2 | 4.9 | **23** |
| Zinc (µg/L) | <100 | <12 | <12 | <12 | <12 | 13 | - | 13 | <12 | 15 | 47 |
| Total hydrocarbons (µg/L) | <50 | 55 | <29 | <29 | 130 | 76 | - | <29 | <29 | 99 | <29 |

Values that exceed the minimum concentration required by Italian law (D.Lgs. 152/2006) are reported in bold type. ORP: Oxidation-reduction potential; DO: Dissolved oxygen; EC: Electrical conductivity.

*For MW-D, 1^st^, 2^nd^, 3^rd^ are referred to the sampling events in three consecutive years.
