# Supplementary material for: 1,2-DCA biodegradation potential of an aquifer assessed in situ and in aerobic and anaerobic microcosms
Source: Environ Microbiome. 2024 Dec 18;19:106. doi: 10.1186/s40793-024-00650-w (PMC11658234; doi:10.1186/s40793-024-00650-w)

Supplementary Material 4. Cladogram built on ARISA profiles of microbial communities from groundwater samples (MWs) from A to H collected in the first sampling campaign and filtered by two different filtration systems (through SFCA or PES membranes). For A-PES sample, ARISA failed. Similarity calculated by Bray-Curtis coefficient.

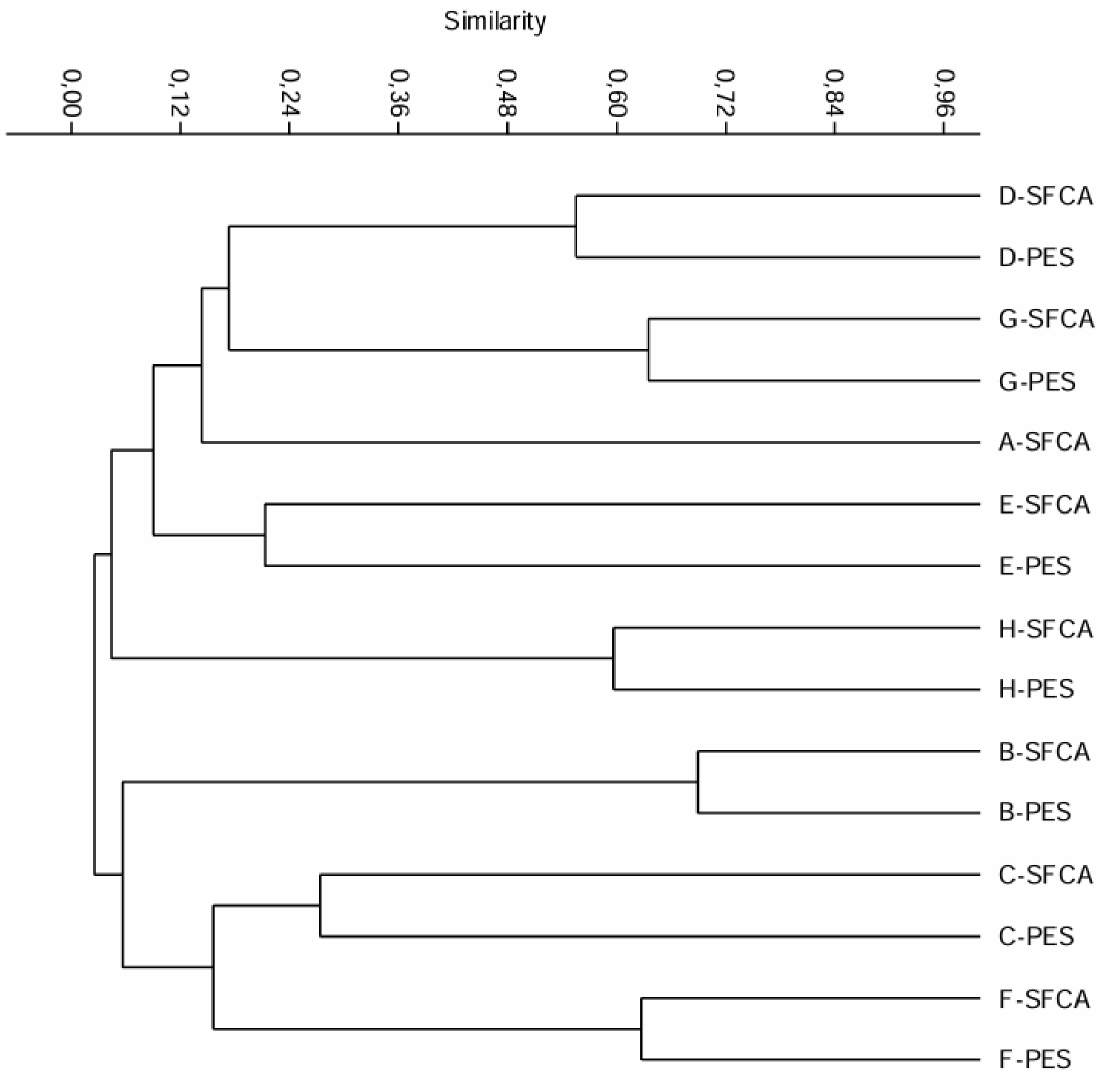

Supplement: Supplementary file 4 — Supplementary Material 4 [file 40793_2024_650_MOESM4_ESM.pdf]
