# Supplementary material for: 1,2-DCA biodegradation potential of an aquifer assessed in situ and in aerobic and anaerobic microcosms
Source: Environ Microbiome. 2024 Dec 18;19:106. doi: 10.1186/s40793-024-00650-w (PMC11658234; doi:10.1186/s40793-024-00650-w)
Supplement: Supplementary file 5 — Supplementary Material 5 [file 40793_2024_650_MOESM5_ESM.docx]

**Supplementary Material 5.** Groundwater microbiota (complete).

|  |  |  |  |  | Relative abundance (%) | | | | | | | | |  |
| --- | --- | --- | --- | --- | --- | --- | --- | --- | --- | --- | --- | --- | --- | --- |
| Phylum | Family | Genus | Dehalogen.metabolism | Ref. | **MW-K** | **MW-A** | **MW-B** | **MW-C** | **MW-D*** | **MW-E** | **MW-F** | **MW-G** | **MW-H** | |
| Actinobacteria | Mycobacteriaceae |  |  |  | nd | 0.31 | nd | 0.10 | 0-0.02 | 0.03 | 0.03 | nd | 0.38 | |
|  |  | ***Mycobacterium*** | DAO, ACo | [2,3] | nd | 0.31 | nd | 0.10 | 0-0.02 | 0.03 | 0.03 | nd | 0.38 | |
| Chloroflexi | Dehalococcoidaceae |  |  |  | 0.14 | nd | 0.60 | 0.06 | 0.26-1.75 | 0.97 | 0.06 | 0.10 | nd | |
|  |  | ***Dehalococcoides*** | RD | [1] | nd | nd | nd | nd | nd^ED^ | nd | nd | nd | nd | |
|  |  | ***Dehalogenimonas*** | RD | [1] | 0.1 | nd | nd | nd | 0.14-1.42^C^ | 0.87 | nd | 0.10 | nd | |
| Firmicutes | Bacillaceae |  |  |  | nd | nd | nd | 0.04 | nd | nd | 0.03 | nd | nd | |
|  |  | ***Bacillus*** | DAO, ACo | [2, 3] | nd | nd | nd | 0.04 | nd | nd | 0.03 | nd | nd | |
| Firmicutes | Peptococcaceae |  |  |  | nd | nd | nd | 0.01 | 0.02-1.80 | 0.60 | 0.03 | 0.08 | nd | |
|  |  | ***Dehalobacter*** | RD | [1] | nd | nd | nd | nd | 0-0.01^C^ | nd | nd | nd | nd | |
|  |  | ***Desulfitobacterium*** | RD | [1] | nd | nd | nd | nd | nd^ED^ | nd | nd | nd | nd | |
|  |  | *Desulfosporosinus* |  |  | nd | nd | nd | nd | 0.02-1.76 | 0.52 | nd | 0.08 | nd | |
|  |  | *Pelotomaculum* |  |  | nd | nd | nd | nd | 0-0.01 | 0.05 | 0.03 | nd | nd | |
| Proteobacteria (α subdivision) | Brucellaceae |  |  |  | nd | 4.49 | nd | 0.10 | nd | nd | nd | nd | nd | |
|  |  | ***Ochrobactrum*** | DAO | [2] | nd | 4.49 | nd | 0.10 | nd | nd | nd | nd | nd | |
| Proteobacteria (α subdivision) | Hyphomicrobiaceae |  |  |  | 0.06 | 0.50 | 0.03 | 0.52 | nd | 0.01 | 0.76 | nd | 0.49 | |
|  |  | *Devosia* |  |  | nd | 0.43 | nd | 0.06 | nd | nd | 0.05 | nd | nd | |
|  |  | ***Hyphomicrobium*** | DAO | [2] | nd | 0.01 | nd | 0.28 | nd | nd | 0.45 | nd | 0.44 | |
|  |  | *Labrenzia* |  |  | 0.02 | nd | nd | nd | nd | nd | nd | nd | nd | |
|  |  | *Parvibaculum* |  |  | 0.04 | nd | 0.03 | 0.05 | nd | 0.01 | nd | nd | 0.05 | |
|  |  | *Pedomicrobium* |  |  | nd | nd | nd | nd | nd | nd | nd | nd | nd | |
|  |  | *Rhodoplanes* |  |  | nd | 0.06 | nd | nd | nd | nd | nd | nd | nd | |
| Proteobacteria (α subdivision) | Methylobacteriaceae |  |  |  | nd | 0.08 | nd | nd | nd | nd | nd | nd | nd | |
|  |  | ***Methylobacterium*** | DAO | [2] | nd | 0.08 | nd | nd | nd | nd | nd | nd | nd | |
| Proteobacteria (α subdivision) | Methylocystaceae |  |  |  | nd | 0.24 | 0.02 | 0.84 | nd | nd | 0.17 | nd | 0.10 | |
|  |  | ***Methylosinus*** | ACo | [3] | nd | nd | 0.02 | 0.81 | nd | nd | 0.17 | nd | nd | |
|  |  | *Pleomorphomonas* |  |  | nd | nd | nd | 0.03 | nd | nd | nd | nd | nd | |
| Proteobacteria (α subdivision) | Xanthobacteraceae |  |  |  | nd | nd | nd | 0.11 | 0-0.01 | 0.02 | nd | 0.02 | nd | |
|  |  | ***Ancylobacter*** | DAO | [7] | nd | nd | nd | nd | nd | nd | nd | 0.02 | nd | |
|  |  | ***Xanthobacter*** | DAO, ACo | [2, 3] | nd | nd | nd | 0.11 | nd | 0.02 | nd | nd | nd | |
| Proteobacteria (β subdivision) | Burkholderiaceae |  |  |  | nd | 0.15 | nd | 0.01 | nd | nd | 0.07 | nd | 0.43 | |
|  |  | ***Burkholderia*** | ACo | [3] | nd | 0.15 | nd | nd | nd | nd | 0.04 | nd | nd | |
| Proteobacteria (β subdivision) | Comamonadaceae |  |  |  | nd | 0.16 | 0.30 | 2.64 | 0-0.03 | 0.03 | 0.78 | 0.02 | 6.15 | |
|  |  | *Acidovorax* |  |  | nd | nd | nd | nd | 0-0.03 | nd | 0.02 | nd | Nd | |
|  |  | *Aquabacterium* |  |  | nd | nd | nd | nd | nd | nd | 0.03 | nd | 0.11 | |
|  |  | ***Hydrogenophaga*** | ACo | [5] | nd | nd | nd | 0.66 | nd | nd | 0.1 | 0.02 | 1.12 | |
|  |  | *Limnohabitans* |  |  | nd | nd | nd | 0.07 | nd | nd | nd | nd | nd | |
|  |  | *Methylibium* |  |  | nd | 0.02 | 0.19 | 0.26 | nd | nd | 0.28 | nd | nd | |
|  |  | *Rhodoferax* |  |  | nd | nd | 0.11 | nd | nd | nd | 0.11 | nd | nd | |
|  |  | *Rubrivivax* |  |  | nd | nd | nd | nd | nd | nd | 0.08 | nd | nd | |
|  |  | *Thiomonas* |  |  | nd | nd | nd | 1.57 | 0-0.01 | 0.03 | 0.02 | nd | nd | |
| Proteobacteria (β subdivision) | Methylophilaceae |  |  |  | 0.17 | nd | 5.71 | 2.63 | nd | nd | nd | nd | 4.46 | |
|  |  | *Methylotenera* |  |  | nd | nd | nd | 2.16 | nd | nd | nd | nd | 0.13 | |
|  |  | ***Methylophilus*** |  |  | nd | nd | nd | nd | nd | nd | nd | nd | 0.1 | |
| Proteobacteria (β subdivision) | Nitrosomonadaceae |  |  |  | nd | nd | nd | nd | nd | nd | 0.76 | nd | 0.24 | |
|  |  | ***Nitrosomonas*** | ACo | [3] | nd | nd | nd | nd | nd | nd | nd | nd | 0.22 | |
| Proteobacteria (ɣ subdivision) | Eubacteriaceae |  |  |  | nd | nd | nd | nd | 0.95-4.52 | 0.15 | nd | 0.17 | N | |
|  |  | ***Acetobacterium*** | CoRD | [4] | nd | nd | nd | nd | 0-0.05 | nd | nd | nd | Nd | |
| Proteobacteria (ɣ subdivision) | Methylococcaceae |  |  |  | 1.2 | nd | 5.53 | 4.99 | nd | nd | 0.43 | nd | 4.91 | |
|  |  | *Methylocaldum* |  |  | nd | nd | nd | nd | nd | nd | 0.03 | nd | Nd | |
|  |  | *Methylomicrobium* |  |  | nd | nd | nd | nd | nd | nd | 0.29 | nd | Nd | |
|  |  | ***Methylomonas*** | ACo | [3] | 0.65 | nd | 2.35 | 3.83 | nd | nd | 0.07 | nd | 1.92 | |
| Proteobacteria (ɣ subdivision) | Pseudomonadaceae |  |  |  | 0.11 | 1.42 | 0.04 | nd | 0.02-0.15 | 0.01 | 0.25 | nd | 0.07 | |
|  |  | ***Pseudomonas*** | CoRD  DAO, ACo | [6]  [3] | 0.11 | 1.04 | nd | nd | nd | nd | 0.1 | nd | 0.07 | |
| Proteobacteria (ɣ subdivision) | Shewanellaceae |  |  |  | 0.04 | nd | nd | nd | 0-0.17 | 0.02 | nd | nd | nd | |
|  |  | ***Shewanella*** | RD | [3] | 0.04 | nd | nd | nd | 0-0.17 | 0.02 | nd | nd | nd | |
| Proteobacteria (ɣ subdivision) | Xanthomonadaceae |  |  |  | 0.09 | 0.69 | 0.61 | 5.13 | 0-0.09 | 0.20 | 0.36 | nd | 0.30 | |
|  |  | *Dyella* |  |  | nd | nd | nd | 0.13 | nd | nd | 0.01 | nd | nd | |
|  |  | *Dokdonella* |  |  | nd | 0.14 | nd | nd | nd | nd | nd | nd | nd | |
|  |  | *Luteimonas* |  |  | nd | nd | nd | nd | nd | 0.03 | nd | nd | nd | |
|  |  | *Pseudoxanthomonas* |  |  | nd | nd | nd | nd | nd | 0.01 | nd | nd | nd | |
|  |  | ***Stenotrophomonas*** | DAO | [2] | nd | 0.47 | 0.01 | nd | 0-0.09 | nd | nd | nd | nd | |
|  |  | *Thermomonas* |  |  | nd | 0.04 | nd | nd | nd | nd | nd | nd | nd | |
| Proteobacteria (δ subdivision) | Desulfovibrionaceae |  |  |  | 0.27 | nd | 0.06 | 0.02 | 0-0.14 | 0.17 | 0.10 | 0.02 | 0.02 | |
|  |  | ***Desulfovibrio*** | RD | [1] | 0.27 | nd | 0.06 | 0.02 | 0-0.14 | 0.17 | nd | 0.02 | 0.02 | |
| Proteobacteria (δ subdivision) | Desulfuromonadaceae |  |  |  | 4.07 | 0.08 | 0.01 | 0.49 | 6.37-13.27 | 0.86 | 0.10 | 1.31 | 0.09 | |
|  |  | ***Desulfuromonas*** | RD | [1] | nd | nd | nd | nd | 0.04-0.23^C^ | nd | nd | nd | nd | |
|  |  | *Desulfuromusa* |  |  | nd | nd | nd | nd | 0-0.58 | nd | nd | nd | nd | |
| Proteobacteria (δ subdivision) | Geobacteraceae |  |  |  | nd | nd | nd | nd | nd | nd | 0.01 | nd | 0.05 | |
|  |  | ***Geobacter*** | RD | [1] | nd | nd | nd | nd | nd | nd | 0.01 | nd | 0.02 | |
| Proteobacteria (δ subdivision) | Syntrophaceae |  |  |  | nd | nd | 0.45 | 0.44 | 0.10-0.93 | 17.01 | 0.53 | 0.02 | 0.13 | |
|  |  | *Desulfobacca* |  |  | nd | nd | 0.40 | 0.16 | 0-0.08 | 15.68 | 0.53 | 0.02 | nd | |
|  |  | ***Desulfomonile*** | RD | [1] | nd | nd | 0.05 | 0.28 | nd | 0.08 | nd | nd | 0.12 | |
| Proteobacteria (ε subdivision) | Campylobacteraceae |  |  |  | 4.01 | 1.40 | nd | 0.71 | 0.04-1.27 | 1.28 | nd | 0.28 | 2.10 | |
|  |  | *Arcobacter* |  |  | 3.29 | 1.40 | nd | 0.71 | 0.02-1.16 | 1.25 | nd | 0.28 | 2.02 | |
|  |  | ***Sulfurospirillum*** | RD | [1] | 0.72 | nd | nd | nd | 0-0.11 | 0.03 | nd | nd | nd | |

Relative abundance of identified bacterial families which include known genera capable of dehalogenating metabolisms of chlorinated solvents (in bold type), in particular direct reductive dechlorination (RD), cometabolic reductive dechlorination (CoRD), direct aerobic oxidation (DAO), or aerobic cometabolism (ACo); other genera identified in each family are also reported. *For MW-D the range of relative abundance referred to the sampling events in three consecutive years is reported. ^ED^: Exclusively detected by PCR probing with OHRB-specific 16S rRNA gene primers; ^C^: Confirmed by PCR probing with OHRB-specific 16S rRNA gene.

1. Türkowsky, D., Jehmlich, N., Diekert, G., Adrian, L., von Bergen, M., & Goris, T. (2018). An integrative overview of genomic, transcriptomic and proteomic analyses in organohalide respiration research. *FEMS microbiology ecology*, *94*(3), fiy013.
2. Xing, Z., Su, X., Zhang, X., Zhang, L., & Zhao, T. (2022). Direct aerobic oxidation (DAO) of chlorinated aliphatic hydrocarbons: A review of key DAO bacteria, biometabolic pathways and in-situ bioremediation potential. *Environment International, 162*, 107165.
3. Dolinová, I., Štrojsová, M., Černík, M., Němeček, J., Macháčková, J., & Ševců, A. (2017). Microbial degradation of chloroethenes: a review. *Environmental Science and Pollution Research, 24(15)*, 13262-13283.
4. De Wildeman, S., Neumann, A., Diekert, G., & Verstraete, W. (2003). Growth-substrate dependent dechlorination of 1, 2-dichloroethane by a homoacetogenic bacterium. *Biodegradation, 14(4)*, 241-247.
5. Lambo, A. J., & Patel, T. R. (2006). Cometabolic degradation of polychlorinated biphenyls at low temperature by psychrotolerant bacterium Hydrogenophaga sp. IA3-A. *Current microbiology*, *53*, 48-52.
6. Fetzner, S. (1998). Bacterial dehalogenation. *Applied microbiology and biotechnology, 50(6)*, 633-657.
7. Kumar, A., Pillay, B., & Olaniran, A. O. (2020). Genome sequence and metabolic analysis revealed the catabolic pathways for the degradation of 1, 2-dichloroethane and other related Xenobiotics in Ancylobacter aquaticus strain UV5. *Gene Reports, 21*, 100969.
